# Along-shelf connectivity and circumpolar gene flow in Antarctic silverfish (*Pleuragramma antarctica*)

**DOI:** 10.1038/s41598-018-36030-x

**Published:** 2018-12-14

**Authors:** Jilda Alicia Caccavo, Chiara Papetti, Maj Wetjen, Rainer Knust, Julian R. Ashford, Lorenzo Zane

**Affiliations:** 1 Department of Biology, University of Padua, Via G. Colombo 3, Padua, 35121 Italy; 2 Consorzio Nazionale Interuniversitario per le Scienze del Mare (CoNISMa), Piazzale Flaminio 9, Rome, 00196 Italy; 3 Institute for Environmental Sciences, University of Koblenz-Landau, Fortstraße 7, Landau, 76829 Germany; 4 Helmholtz Center for Polar and Marine Research, Alfred Wegener Institute, Am Alten Hafen 26, Bremerhaven, 27568 Germany; 5 Department of Ocean, Earth and Atmospheric Sciences, Center for Quantitative Fisheries Ecology, Old Dominion University, 800 West 46th Street, Norfolk, VA 23508 United States

## Abstract

The Antarctic silverfish (*Pleuragramma antarctica*) is a critically important forage species with a circumpolar distribution and is unique among other notothenioid species for its wholly pelagic life cycle. Previous studies have provided mixed evidence of population structure over regional and circumpolar scales. The aim of the present study was to test the recent population hypothesis for Antarctic silverfish, which emphasizes the interplay between life history and hydrography in shaping connectivity. A total of 1067 individuals were collected over 25 years from different locations on a circumpolar scale. Samples were genotyped at fifteen microsatellites to assess population differentiation and genetic structuring using clustering methods, *F*-statistics, and hierarchical analysis of variance. A lack of differentiation was found between locations connected by the Antarctic Slope Front Current (ASF), indicative of high levels of gene flow. However, gene flow was significantly reduced at the South Orkney Islands and the western Antarctic Peninsula where the ASF is absent. This pattern of gene flow emphasized the relevance of large-scale circulation as a mechanism for circumpolar connectivity. Chaotic genetic patchiness characterized population structure over time, with varying patterns of differentiation observed between years, accompanied by heterogeneous standard length distributions. The present study supports a more nuanced version of the genetic panmixia hypothesis that reflects physical-biological interactions over the life history.

## Introduction

### Silverfish life history and connectivity

The Antarctic silverfish (*Pleuragramma antarctica*) is a critically important forage species in the Southern Ocean that connects higher and lower trophic levels in the continental shelf ecosystem^1,2^. Having a circumpolar distribution, silverfish dominate the high-Antarctic neritic assemblage in terms of both biomass and abundance^3^. Atypical for a notothenioid, silverfish have a wholly pelagic life cycle, including a cryopelagic egg and larval phase^4^. Silverfish have adopted a relatively inactive, energy-conserving life strategy similar to related benthic species, in which their neutral buoyancy allows them to remain suspended in the water column without active swimming^3,5^. Remaining in the water column throughout their life history exposes silverfish to current and front systems over the slope and shelf that have been hypothesized by Ashford *et al*.^6^ to play an integral role in shaping their circumpolar distribution^6^.

**Figure 1:**
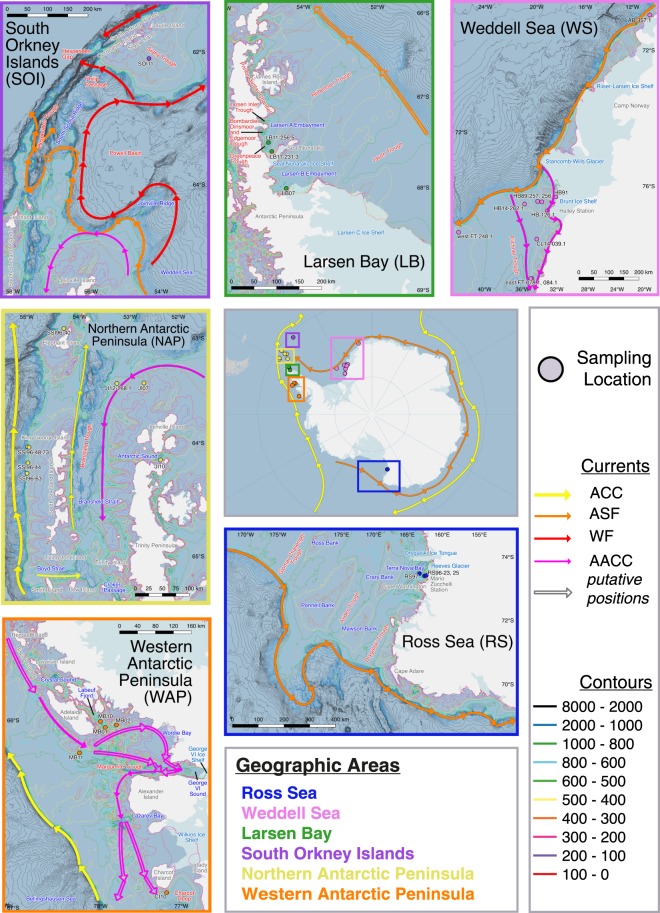
Sampling locations. **Overview** Map of the Antarctic, showing sampling areas. Sampling locations are color-coded by geographic region. Areas of interest in subsequent maps are indicated in color-coded squares and ordered by movement along the ASF. Inferred position of the ACC and ASF approximated based on Fig. 7 in Orsi *et al*.^11^ and Whitworth *et al*.^8^, respectively. **Ross Sea (RS)** Position of the ASF is approximated based on Ashford *et al*.^6^ Fig. 10.11. **Weddell Sea (WS)** Position of the AACC and ASF is approximated based on Fig. 2b in Nicholls *et al*.^56^. **Larsen Bay (LB)** Putative position of the ASF according to Whitworth *et al*.^8^. **Northern Antarctic Peninsula (NAP)** Position of the ACC and AACC are based on Fig. 7 in Orsi *et al*.^11^ and Fig. 3 in Thompson *et al*.^47^, respectively. Entry of Circumpolar Deep Water from the ACC into the Bransfield Strait through the Boyd Strait is shown according to Savidge and Amft^10^. Yellow and pink arrows within the Bransfield Strait and Trough area represent components of the Bransfield Gyre with Bellingshausen influence and Weddell influence respectively, based on Fig. 15 in Sangrà *et al*.^87^. **South Orkney Islands (SOI)** Schematic illustration of the circulation drawn from: ACC (Fig. 7 in Orsi *et al*.^11^), ASF (Whitworth *et al*.^8^, Fig. 6 in Heywood *et al*.^9^, Fig. 14 in Azaneu *et al*.^51^), AACC (Fig. 3 in Thompson *et al*.^47^, Fig. 15 in Sangrà *et al*.^87^), and WF (Fig. 6 in Heywood *et al*.^9^, Fig. 3 in Thompson *et al*.^47^, Fig. 14 in Azaneu *et al*.^51^). Putative position of the ASF according to Azaneu *et al*.^51^. **Western Antarctic Peninsula (WAP)** Position of the ACC and AACC is approximated based on both, Fig. 7 in Orsi *et al*.^11^ and Fig. 14 in Moffat *et al*.^7^ respectively. Empty arrows indicate the putative position of the AACC according to Moffat *et al*.^7^. ACC, Antarctic Circumpolar Current; ASF, Antarctic Slope Front; WF, Weddell Front; AACC, Antarctic Coastal Current. Maps created using the Norwegian Polar Institute’s Quantarctica 2.0 package^88^ in the software QGIS version 2.18.9 http://qgis.osgeo.org ^89^.

The silverfish life history hypothesis predicts along-shelf connectivity facilitated principally by three major features of the large-scale circulation. The Antarctic Coastal Current (AACC) is typical of coastal buoyant plumes^7^ and transports water westward between glacial trough systems along the inner shelf. Similarly, a horizontal pressure gradient across the Antarctic Slope Front (ASF) drives westward transport in the Antarctic Slope Front Current. In this paper, ASF refers to this system, including the Antarctic Slope Front Current. It is located over the continental slope and thought to be continuous from the Amundsen Sea along the Ross Sea continental shelf and East Antarctica, forming the southern limb of the Weddell Gyre and reaching waters north of the Antarctic Peninsula^8,9^ (Fig. 1, overview). In contrast to the AACC and ASF, the Antarctic Circumpolar Current (ACC) transports water eastward. It approaches the continental shelf in the Amundsen Sea where it bifurcates, and its southern boundary is located over the slope off the western Antarctic Peninsula before moving seaward west of the South Shetland Islands^10,11^ (Fig. 1, overview).

Entrainment of silverfish in transport pathways is facilitated by a life history characterized by vertical migrations associated with different life stages^12^. Eggs and larvae are found from 0–100 m among the platelet ice layer beneath coastal fast ice^13^. Descent to deeper layers begins at the post-larval phase, and post-larvae and juveniles up to 2 + years are found at depths up to 400 m^3^. Adult silverfish, ranging in length between approximately 8–25 cm, are typically found at depths from 400–700 m, exhibiting a diel migration in which a greater abundance of fish are present in the shallower portion of their distribution by night, and in deeper waters by day^3,14^. These changes in habitat occupancy correlate with ontogenetic shifts in diet composition, such that silverfish occupy several trophic levels throughout their 12–14 year lifespan^3,15^. While feeding almost exclusively on zooplankton, silverfish have been shown to exhibit dietary plasticity, including cannibalism^16–18^. Thus, early-life dependence on coastal sea-ice zones and later movement into deeper waters in pursuit of prey and avoidance of predators expose fish to hydrographic features along the continental shelf. This results in a complex life history which generates the conditions for a dynamic population structure and underlying patterns of connectivity^6^.

The silverfish life history hypothesis incorporates these physical-biological interactions, predicting that cross-shelf circulation mediates retention within populations and exposure to along-shelf transport pathways^6,19,20^. Schools of adult silverfish have been observed moving inshore along the Antarctic Peninsula^21^. Assemblages of silverfish eggs and larvae have been found in the summer polynya of the eastern Weddell Sea^22^, in waters along the Antarctic Peninsula^23^, under sea ice in the western Ross Sea^4,12,24^, and in the Dumont d’Urville Sea^25^ (see Fig. 1 for place names). These observations suggest a life cycle in which adults return to coastal areas each winter to spawn. This migration is facilitated by the circulation within glacial trough systems across the continental shelf. While trough outflows aid in the dispersal of developing fish away from hatching sites, inflows function to retain adults by transporting them back to spawning areas. The discovery of newly hatched larvae in trough systems along the Weddell Sea, Ross Sea, and Antarctic Peninsula^6,13,22,26^ implies this cycle of dispersion and retention occurs in multiple areas around the Antarctic. However, such a cycle would be vulnerable to advective losses along the shelf in the AACC, and along the slope in the ASF or ACC, resulting in transport to locations downstream^6^.

### Genetic structuring and gene flow around the Antarctic

Estimates of genetic differentiation are a principal tool for defining population structure, by assessing gene flow between populations, as well as the extent to which inbreeding and genetic drift contribute to population differentiation^27^. The first hypotheses concerning population structure in silverfish arose from research carried out in the 1980s in the eastern Weddell Sea and Antarctic Peninsula^22,23^. Recent advances in our understanding of hydrography and physical-biological interactions provide a contemporary life history framework in which the population genetic structure of silverfish needs to be considered (see Ashford *et al*.^6^).

Highly connected populations resulting in genetic panmixia have previously been the null hypothesis to describe genetic structuring in notothenioids. This hypothesis is based on the expected influence of circumpolar currents on the transport of pelagic larvae, which would sustain high levels of gene flow^28^. This was supported by genetic homogeneity found among multiple species of the families Nototheniidae and Channichthyidae inhabiting the Scotia Arc^29,30^. At the same time however, this panmictic hypothesis was challenged by evidence of population structuring based on genetic heterogeneity in multiple other species, including *Dissostichus mawsoni*^31,32^, *Chionodraco hamatus*^33^, *Chaenocephalus aceratus*^34,35^, and *Champsocephalus gunnari*^35^.

Evidence for gene flow and genetic panmixia has been similarly variable for silverfish. Using mitochondrial DNA markers, Zane *et al*.^36^ found little indication of population structuring in silverfish on a circumpolar scale, although differences were found between sampling years in the eastern Weddell Sea, and between one sample in the eastern Weddell Sea and one of two taken in the western Ross Sea^36^. A more recent study on silverfish connectivity around the Antarctic Peninsula employing microsatellite markers revealed that fish taken from Marguerite Bay and Charcot Island along the western Antarctic Peninsula (WAP) were significantly different from those along the northern Antarctic Peninsula (NAP) at Joinville Island and on the eastern side of the peninsula at Larsen Bay (LB)^19^ (see Fig. 1, WAP, NAP, and LB for place names). Samples were collected over multiple years, and as in Zane *et al*.^36^, evidence for restrictions to gene flow varied between years^19^.

Variation in recruitment and dispersal, which could explain the observed changes in silverfish distribution, is predicted by the hypothesis of chaotic genetic patchiness^37^, developed to explain the fluctuations in gene flow between sampling years^19^. Current theory emphasizes the role of physical-biological interactions in structuring populations and the potential for extensive along-shelf connectivity. Yet to date, there has been no comprehensive investigation of silverfish gene flow and population structure at different spatial scales and over time^6^, nor re-examination of earlier genetic evidence in the context of recent theoretical advances in understanding.

The present study builds upon the Matschiner *et al*.^28^ hypothesis of genetic panmixia in the light of the physical-biological framework provided by Ashford *et al*.^6^. It aims to test for population structuring and along-shelf connectivity mediated by large-scale circulation. Employing microsatellite markers, we assessed gene flow between silverfish sampled over 25 years from six different geographic regions around Antarctica (Table 1). The following regions were investigated based on observed and predicted silverfish larval assemblages: the western Ross Sea^4,12,24^, eastern Weddell Sea^22^, Larsen Bay^26^, northern Antarctic Peninsula^23^, South Orkney Islands^38^, and western Antarctic Peninsula^39^. Inclusion of samples from previous studies allowed for a greater number of spatial and temporal comparisons. The expansion of the Agostini *et al*.^19^ analysis of Antarctic Peninsular connectivity enabled comparisons to be made on a circumpolar scale. Finally, the use of microsatellite markers increased the analytical power of the Zane *et al*.^36^ mitochondrial DNA-based study^40^. With the present approach, we were able to compare shelf areas located along the ASF from the Ross Sea to the tip of the Antarctic Peninsula, as well as areas located along the ACC from Charcot Island to the South Shetland Islands. In addition, we tested for potential gene flow downstream to the South Orkney Islands along the southern ACC and Weddell Gyre. Finally, standard length (SL) data from over half of the samples collected indicated differences in SL distributions between sampling locations, and we used these to test for chaotic genetic patchiness. Where more than one SL mode existed, sampling locations were further divided into groups of smaller and larger fish to examine for genetic differentiation between and among different cohorts. Although temporal replicates differed between sampling locations, and more locations were sampled in some geographic regions than others, the dataset allowed us to test for genetic panmixia on a circumpolar scale for the first time in silverfish.

**Table 1:** *Pleuragramma antarctica* sampling locations between 1989 and 2014 from the six geographic regions analyzed in this study.

Sampling location	Year	Acronym	Campaign	Coordinates		*n*
**Western Antarctic Peninsula (WAP)**						**280**
Charcot Island (CI)	2010	CI10	NBP 10–02^a^	−70.117	−76.033	60
Marguerite Bay (MB)	2001	MB01	SO GLOBEC-Cruise 1^b^	−67.950	−68.350	28
	2002	MB02	SO GLOBEC-Cruise 3^b^	−68.133	−68.017	49
	2010	MB10	NBP 10–02^a^	−67.817	−68.150	60
	2011	MB11	LMG Cruise 11–01, Palmer LTER^c^	−67.650	−70.067	83
**Northern Antarctic Peninsula (NAP)**						**250**
Joinville Island (JI)	2007	JI07	ANT-XXIII/8^d^	−62.583	−54.750	34
	2010	JI10	NBP 10–02^a^	−63.500	−56.667	148
	2012	JI12	ANT-XXVIII/4^d^	−62.233	−55.300	54
South Shetland Islands (SSI)	1996	SSI96	ANT-XIV/2^d^	−61.549	−58.205	14
**South Orkney Islands (SOI)**	2011	SOI11	ANT-XXVII/3^d^	−61.179	−45.673	**47**
**Larsen Bay (LB)**						**98**
	2007	LB07	ANT-XXIII/8^d^	−65.500	−61.667	46
	2011	LB11	ANT-XXVII/3^d^	−64.833	−60.350	52
**Weddell Sea (WS)**						**217**
Filchner Trough (FT)	2014	FT14	ANT-XXIX/9^d^	−76.217	−34.961	50
Halley Bay (HB)	1989	HB89	ANT-VII/4^d^	−75.155	−27.788	19
	1991	HB91	ANT-IX/3^d^	−75.250	−25.835	41
	2014	HB14	ANT-XXIX/9^d^	−75.452	−28.703	82
Atka Bay (AB)	2014	AB14	ANT-XXIX/9^d^	−70.913	−10.735	25
**Ross Sea (RS)**						**175**
Terra Nova Bay	1996	RS96	11th Italian expedition PNRA Italica^e^	−74.810	164.303	91
	1997	RS97	13th Italian expedition PNRA Italica^e^	NA		84

Sampling location, year, acronym, campaign, coordinates, and sample size (*n*) are indicated.

^a^Research vessel (RV) *Nathaniel B. Palmer*. Collection via Multiple Opening and Closing Net, with an Environmental Sensing System (10 m^2^ MOCNESS, MOC-10) outfitted with six 3 mm mesh nets^19,50,90,91^.

^b^Antarctic Research Support Vessel (ARSV) *Laurence M. Gould* as part of the Southern Ocean Global Ocean Ecosystems Dynamics (SO GLOBEC) program. Collection via 10 m^2^ MOCNESS, MOC-10, outfitted with six 3 mm mesh nets^19,50,90,91^.

^c^ARSV *Laurence M. Gould* as part of the Palmer Long-Term Ecological Research (LTER) program. Collection via 2 × 2 m square-frame net with a 700 µm mesh^92^.

^d^RV *Polarstern*, Alfred Wegner Institute for Polar Research (AWI), Bremerhaven, Germany. Collection via commercial benthopelagic trawl, with a cod-end mesh line of 20 mm^36^.

^e^RV *Italica* as part of the National Program of Research in Antarctica (PNRA), Italy. Collection via Hamburg Plankton net with 0.5–1 mm cod-end^36^.

## Results

### Genotyping

Genotypes from 16 microsatellite loci were successfully obtained for all 505 newly sequenced individuals, further supporting that microsatellites developed for *C. hamatus*^19^ can be used in *P. antarctica*. While amplifying successfully in all individuals, one locus (Ch11230) was problematic in terms of accurate genotyping, due to stuttering and interference from the other fluorophores used to detect fragments in sequencing. Subsequent analyses were carried out on both datasets, including and excluding this locus, and no major differences were obtained in the results. Thus, results are presented based on 15 microsatellite loci, excluding locus Ch11230, for verisimilitude. No significant linkage disequilibrium was observed between loci. Significant departures from Hardy Weinberg Equilibrium (HWE) were found at 22 of 304 tests after SGoF + correction for multiple tests (threshold for significance with 304 comparisons *P* = 0.008) with no locus exhibiting Hardy Weinberg Disequilibrium (HWD) in more than 3 sampling locations and no sampling location containing more than 5 loci in HWD (Table S1). Of the 20 occasions in which the software Micro-Checker found evidence for null alleles at a locus in a given sampling location, only 3 of those coincided with an instance of HWD. Thus, it is likely that the 22 instances of HWD were not representative of an artifactual excess of homozygotes among sampling locations for the loci tested. Evidence for overall heterozygosity excess (*H*_E_ > *H*_O_) was not found by population or by locus, though greater numbers of loci within populations displayed extreme excesses of *H*_E_ than such excesses of *H*_O_ (27 comparisons with *H*_E_ exceeding *H*_O_ by > 0.1 versus 13 comparisons with *H*_O_ exceeding *H*_E_ by > 0.1, out of 304 comparisons). However, only 2 of the 27 instances of extreme *H*_E_ excess coincided with a significant instance of HWD. Thus, given that the effect size is quite small for the majority (20 of 22) of the instances of HWD, it is unlikely that these departures from HWE are of biological importance^41^. Neither ARLEQUIN nor LOSITAN identified any locus as a putative outlier, with all simulated *F*_ST_ values falling within neutral expectations. High levels of genetic variation were observed in comparable values of *N*_A_*, A*_R_*, H*_O_, and *H*_E_ (Table 2, Table S1), showing no significant differences (one-way ANOVA, *P* > 0.05) across all sampling locations.

**Table 2:** Summary of genetic variability for *Pleuragramma antarctica* from 19 sampling locations at 15 microsatellite loci.

Sampling location	*n*	*N*_A_ ± SD	*A*_R_ ± SD	*H*_O_ ± SD	*H*_E_ ± SD	*pHWE*
CI10	60	8.40 (5.01)	5.61 (3.13)	0.58 (0.20)	0.62 (0.21)	0.288
MB01	28	7.46 (4.70)	5.54 (3.09)	0.61 (0.27)	0.61 (0.24)	0.766
MB02	49	8.20 (4.27)	5.54 (2.91)	0.62 (0.24)	0.61 (0.23)	0.632
MB10	60	8.86 (5.08)	5.93 (2.94)	0.65 (0.21)	0.64 (0.21)	0.059
MB11	83	9.26 (5.39)	5.75 (3.05)	0.62 (0.22)	0.62 (0.23)	0.773
JI07	34	7.60 (4.74)	5.52 (2.85)	0.63 (0.22)	0.63 (0.21)	0.702
JI10	148	11.00 (5.75)	6.02 (2.85)	0.64 (0.19)	0.65 (0.20)	**0.012**
JI12	54	8.00 (4.05)	5.45 (2.65)	0.60 (0.22)	0.62 (0.21)	0.044
SSI96	14	5.86 (3.39)	4.98 (2.65)	0.60 (0.22)	0.60 (0.22)	0.077
SOI11	47	7.93 (3.57)	5.59 (2.47)	0.60 (0.19)	0.63 (0.18)	0.080
LB07	46	7.80 (4.31)	5.49 (2.75)	0.61 (0.24)	0.62 (0.22)	**0.011**
LB11	52	8.53 (4.37)	5.75 (2.92)	0.63 (0.22)	0.63 (0.22)	0.690
FT14	50	8.20 (3.74)	5.70 (2.62)	0.62 (0.21)	0.63 (0.21)	0.909
HB89	19	6.13 (2.61)	5.12 (2.06)	0.62 (0.20)	0.63 (0.19)	0.095
HB91	41	7.93 (4.35)	5.66 (2.81)	0.62 (0.23)	0.63 (0.21)	0.274
HB14	82	9.26 (4.36)	5.69 (2.79)	0.60 (0.19)	0.63 (0.20)	0.037
AB14	25	6.73 (3.12)	5.24 (2.44)	0.64 (0.23)	0.61 (0.22)	0.026
RS96	91	9.80 (4.82)	5.93 (2.94)	0.63 (0.21)	0.64 (0.21)	0.209
RS97	84	9.00 (5.19)	5.73 (2.94)	0.62 (0.20)	0.63 (0.20)	0.913

Sample size (*n*), number of alleles (*N*_A_), allelic richness (*A*_R_), observed heterozygosity (*H*_O_), unbiased heterozygosity (*H*_E_), and probability of deviation from Hardy-Weinberg equilibrium (*pHWE*) are shown. Allelic richness is calculated based on a minimum sample size of 14 individuals. Standard deviation ( ± SD) is given in parentheses. Values in bold indicate significant HWE deviations after correction for multiple tests as implemented in SGoF + (threshold for significance with 285 comparisons *P* = 0.0137). Sampling location acronyms are as in Table 1.

### Population structure

A power analysis showed that the 15 microsatellites employed in the analysis of genetic differentiation had a 100% chance to detect *F*_ST_ values above 0.0025. The analysis was performed considering various combinations of observed allelic frequencies and sample sizes at multiple iterations of effective population size (N_e_) (1000–10,000) and generations (2–201) (Table S4). For *F*_ST_ values greater than 0.001, there was a 94.1–97.4% chance of detecting significant *F*_ST_ values for *t* = 2, 10, 20 and N_e_ = 1000, 5000, 10000 using both Fisher’s and Chi-square tests. Thus, the present dataset has the power to detect differences associated with *F*_ST_ higher than 0.001. In the current study, all significant comparisons obtained for *F*_ST_ estimates ranged between 0.00388 and 0.0125, and all near-significant values (for which 0.1 > *P* > 0.03326, the threshold for significance after SGoF + correction) ranged from 0.00168 to 0.00572. This confirms that all non-significant comparisons can be presumed to be such for the present dataset, and are not non-significant due to the inability of the markers used to detect differences among samples.

Hierarchical AMOVA (Table S3) indicated that partitioning the 19 sampling locations into *k* = 6 groups based on geographic region (Ross Sea (RS), Weddell Sea (WS), Larsen Bay (LB), northern Antarctic Peninsula (NAP), South Orkney Islands (SOI), and western Antarctic Peninsula (WAP), Table 1) maximized between group heterogeneity (global *F*_CT_ = 0.00159; *P* = 0.00218 ± 0.00050) while minimizing within group heterogeneity (global *F*_sc_ = 0.00002; *P* = 0.63050 ± 0.00429). Genetic differentiation was present (global *F*_ST_ = 0.00161; *P* = 0.00644 ± 0.00087) but very low, with <1% of the total variation due to sampling location. Only 0.16% of variation was attributed to variation among groups. Locus-by-locus AMOVA confirmed that no particular locus had a significantly outsized impact on the calculation of global *F*-statistics.

**Figure 2:**
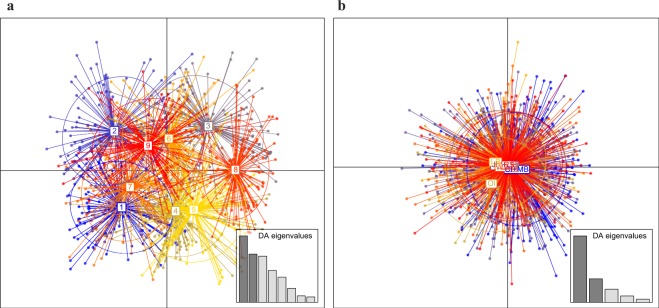
Discriminant analysis of principal components (DAPC) clustering results for *Pleuragramma antarctica* populations. (**a**) DAPC run without *a priori* parameters. (**b**) DAPC run based on geographic region, acronyms are as in Table 1.

**Figure 3:**
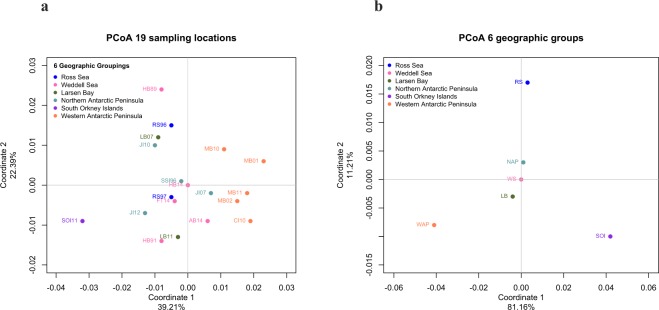
Principal coordinate analysis (PCoA) for *Pleuragramma antarctica* samples based on *F*_ST_ genetic distance. (**a**) PCoA of 19 sampling locations. (**b**) PCoA of six geographic groups. Sampling location and geographic group acronyms are as in Table 1.

Clustering methods failed to indicate a clear structure among the sampling locations analyzed. Clustering methods employed by DAPC and FLOCK (without pre-defined groups) randomly assigned individuals to groups, with no correlation to sampling location or geographic region (Fig. 2, S1). DAPC run without *a priori* parameters suggested the existence of nine clusters (Fig. 2a), with *k* = 9 selected based on cluster optimization plotted against Bayesian Information Criteria (Fig. S2a). The same DAPC based on the six geographic regions (RS, WS, LB, NAP, SOI, and WAP), run in an attempt to maximize variation between localities, showed no evidence of cluster formation (Fig. 2b). PCoA on all 19 sampling locations provided limited support for geographic clustering: the first principal coordinate accounted for 39.21% of the variance and clearly distinguished the SOI and WAP samples from all others (Fig. 3a). PCoA on the six geographic regions (RS, WS, LB, NAP, SOI, and WAP) emphasized this delineation further: the first principal coordinate accounted for 81.16% of the variance, and separated the SOI and WAP samples from the rest (Fig. 3b). However, in both cases PCoA failed to distinguish NAP, WS, and LB from one another, and also from the other geographic regions.

In contrast to clustering methods, pairwise *F*_ST_ provided support for geographic population clusters. Within sampling locations, there were no significant differences between samples collected over multiple years (*F*_ST_ ranged from −0.0025 to 0.0024, *P* > 0.05 for all comparisons, Table 3), suggestive of genetic stability over time. In addition, within geographic regions (RS, WS, LB, NAP, SOI, and WAP), there was no significant difference between sampling locations (*F*_ST_ ranged from −0.0051 to 0.0024, *P* > 0.05 for all comparisons, Table 3), indicative of genetic homogeneity within these regions.

**Table 3:** Genetic differentiation among *Pleuragramma antarctica* samples based on microsatellite data.

	Western Antarctic Peninsula (WAP)					Northern Antarctic Peninsula (NAP)				SOI	Larsen Bay (LB)		Weddell Sea (WS)					Ross Sea (RS)	
	CI10 (60)	MB01 (28)	MB02 (49)	MB10 (60)	MB11 (83)	JI07 (34)	JI10 (148)	JI12 (54)	SSI96 (14)	SOI11 (47)	LB07 (46)	LB11 (52)	FT14 (50)	HB89 (19)	HB91 (41)	HB14 (82)	AB14 (25)	RS96 (91)	RS97 (84)
**CI10**	—	−0.0008	−0.0006	0.0007	−0.0008	−0.0033	**0.0042**	0.0043	−0.0032	**0.0100**	**0.0054**	0.0021	0.0002	0.0045	0.0016	0.0001	−0.0012	**0.0049**	0.0024
**MB01**	0.6781	—	−0.0008	−0.0022	−0.0016	0.0006	0.0043	0.0047	0.0013	**0.0125**	0.0057	0.0057	0.0007	0.0010	0.0037	0.0015	0.0008	0.0043	0.0042
**MB02**	0.6476	0.5735	—	0.0009	0.0008	−0.0014	0.0017	0.0043	−0.0027	**0.0100**	0.0024	0.0001	0.0008	0.0032	−0.0003	−0.0008	−0.0043	0.0019	**0.0039**
**MB10**	0.3761	0.8023	0.2933	—	0.0009	−0.0037	0.0009	0.0037	0.0015	**0.0091**	0.0027	−0.0005	−0.0000	−0.0030	0.0008	−0.0011	−0.0021	0.0002	0.0010
**MB11**	0.7148	0.7278	0.2908	0.2391	—	−0.0008	**0.0040**	**0.0043**	0.0014	**0.0096**	**0.0094**	**0.0046**	0.0008	0.0019	0.0030	0.0018	−0.0001	**0.0041**	0.0025
**JI07**	0.9578	0.4125	0.6796	0.9656	0.6136	—	−0.0010	0.0010	−0.0046	**0.0059**	0.0009	−0.0034	−0.0034	0.0003	0.0002	−0.0027	−0.0053	−0.0018	−0.0026
**JI10**	**0.0050**	0.0468	0.1351	0.2255	**0.0009**	0.7206	—	0.0018	−0.0019	0.0031	0.0021	0.0000	−0.0001	−0.0040	0.0007	−0.0004	0.0001	−0.0010	0.0017
**JI12**	0.0376	0.0844	0.0388	0.0389	**0.0117**	0.3609	0.1218	—	0.0011	0.0013	0.0039	0.0027	−0.0006	0.0023	−0.0015	0.0015	0.0014	0.0020	0.0020
**SSI96**	0.8883	0.3942	0.7829	0.3009	0.3053	0.8963	0.7773	0.4223	—	0.0013	0.0022	−0.0023	−0.0032	−0.0068	−0.0018	−0.0035	−0.0034	−0.0005	0.0002
**SOI11**	**0.0003**	**0.0019**	**0.0004**	**0.0001**	**<0.0001**	**0.0333**	0.0398	0.3352	0.4097	—	**0.0058**	0.0039	0.0003	0.0028	−0.0001	0.0037	0.0049	**0.0066**	0.0031
**LB07**	**0.0194**	0.0552	0.1438	0.0998	**<0.0001**	0.3566	0.1040	0.0585	0.2782	**0.0235**	—	0.0020	0.0020	−0.0016	**0.0056**	0.0004	0.0004	0.0033	0.0033
**LB11**	0.1878	0.0417	0.4465	0.5802	**0.0083**	0.9410	0.4981	0.1197	0.7620	0.0630	0.1823	—	−0.0012	0.0066	−0.0012	−0.0008	−0.0035	0.0016	0.0013
**FT14**	0.5132	0.4165	0.3355	0.4850	0.3013	0.9320	0.5431	0.6490	0.8470	0.4840	0.1973	0.7401	—	−0.0012	−0.0012	−0.0015	−0.0037	−0.0009	−0.0013
**HB89**	0.2861	0.4788	0.2574	0.7111	0.3197	0.4860	0.8371	0.3928	0.8850	0.3797	0.6357	0.1285	0.6179	—	0.0021	−0.0025	0.0016	−0.0048	0.0003
**HB91**	0.2813	0.1429	0.5330	0.3281	0.0726	0.4670	0.3548	0.7993	0.7201	0.5527	**0.0251**	0.7284	0.7284	0.3960	—	−0.0006	−0.0031	0.0015	0.0007
**HB14**	0.5533	0.3417	0.7140	0.8004	0.1089	0.9379	0.7097	0.2525	0.9270	0.0534	0.4498	0.7405	0.8699	0.7597	0.6806	—	−0.0051	0.0011	−0.0009
**AB14**	0.6856	0.3856	0.9274	0.7509	0.4464	0.9520	0.4831	0.3312	0.7616	0.0891	0.4093	0.9015	0.9162	0.3727	0.8489	0.9932	—	0.0011	−0.0004
**RS96**	**0.0047**	0.0512	0.1306	0.4294	**0.0026**	0.8272	0.9078	0.1130	0.5626	**0.0001**	0.0391	0.1572	0.7323	0.8800	0.2032	0.2109	0.3073	—	0.0024
**RS97**	0.0939	0.0758	**0.0274**	0.2540	0.0365	0.9262	0.0674	0.1339	0.4861	0.0740	0.0520	0.2238	0.8167	0.5103	0.3830	0.8084	0.5485	0.0398	—

Pairwise *F*_ST_ estimates (above the diagonal) and corresponding *P*-values (below the diagonal) are shown. Values in bold were significant after correction for multiple tests as implemented in SGoF + (threshold for significance with 171 comparisons *P* = 0.0332). Comparisons within the same geographic region are delineated with dotted lines. Numbers in parentheses adjacent to population sample names specify the sample size (*n*). Sampling location acronyms are as in Table 1. SOI, South Orkney Islands.

Pairwise comparisons between years and different sampling locations among the geographic groupings displayed limited structure. Patterns of significant differentiation previously found along the Antarctic Peninsula were maintained to some extent (5 significant comparisons out of 36 in the current study and 9 out of 36 in Agostini *et al*.^19^ after SGoF + correction for multiple tests, threshold for significance with 171 comparisons *P* = 0.0332). Such discrepancies are due to the use of 15 loci instead of 16 loci as in Agostini *et al*.^19^ as well as to changes in significance thresholds resulting from the increased number of comparisons in the present study (nineteen sampling locations versus nine). Indeed, when the dataset of nine sampling locations from Agostini *et al*.^19^ was reanalyzed with the removal of the problematic locus Ch11230, three differences were found with respect to the original analysis (Table S5). Two of these disparities from the original analysis (the comparisons between CI10 and LB07, and between MB01 and JI12) were also seen in the larger analysis between nineteen sampling locations (Table 3). Variation in differentiation between years in comparisons from the same sampling location, as previously documented in the Ross Sea^36^ and the Antarctic Peninsula^19^, was also obtained in the current study. For every location for which samples existed from multiple years, patterns of differentiation varied over sampling years (Table 3). This was especially evident at Marguerite Bay, Joinville Island, the South Orkney Islands, Larsen Bay, and the Ross Sea.

Grouping sampling locations by geographic region provided evidence of weak genetic structuring (*F*_ST_ ranged from 0.0001 to 0.0153, Table 4). Significant pairwise *F*_ST_ values were found between all groups and the western Antarctic Peninsula group (also seen in Agostini *et al*.^19^), as well as between the South Orkney Islands and all groups except for the Weddell Sea, where the comparison was not significant after SGoF + correction for multiple tests (threshold for significance with 15 comparisons *P* = 0.0142, Table 4). In addition, the comparison between Larsen Bay and the Ross Sea was not significant after correction (*P* = 0.0312, Table 4).

**Table 4:** Genetic differentiation among *Pleuragramma antarctica* samples, pooling sampling locations from common geographic regions.

	WAP (280)	NAP (257)	SOI (47)	LB (98)	WS (210)	RS (175)
**WAP**	—	**0.0039**	**0.0153**	**0.0041**	**0.0034**	**0.0059**
**NAP**	**<0.0001**	—	**0.0039**	0.0005	0.0001	0.0001
**SOI**	**<0.0001**	**0.0079**	—	**0.0056**	0.0038	**0.0049**
**LB**	**<0.0001**	0.2865	**0.0033**	—	0.0007	0.0017
**WS**	**<0.0001**	0.4538	0.0143	0.2067	—	0.0001
**RS**	**<0.0001**	0.4597	**0.0029**	0.0312	0.4492	—

Pairwise *F*_ST_ estimates (above the diagonal) and corresponding *P*-values (below the diagonal) are shown. Values in bold were significant after correction for multiple tests as implemented in SGoF + (threshold for significance with 15 comparisons *P* = 0.0142). Numbers in parentheses adjacent to group names indicate the sample size (*n*). Group acronyms are as in Table 1.

### Comparison by length modes

Of the 1067 samples analyzed in this study, standard length (SL) data were available for 671 (63%) samples. SL ranged from 3.7–21.5 cm. Post-larvae and juveniles (SL < 9 cm)^3^ composed 15% of all fish for which SL data were available. To control for the possibility of bias presented by the wide range of SL represented in the present study, both DAPC and pairwise *F*_ST_ analyses were performed on SL-derived groups independent of sampling location. Six groupings were tested, separating fish by SL into groups divided by 1, 2, 3, 4, 5, and 6 cm increments (Table S6a). No evidence of clustering was seen in the DAPC analysis of the length groupings (Fig. S3), nor was any pattern of genetic differentiation associated with life stage observed (Table S6b–g).

**Figure 4:**
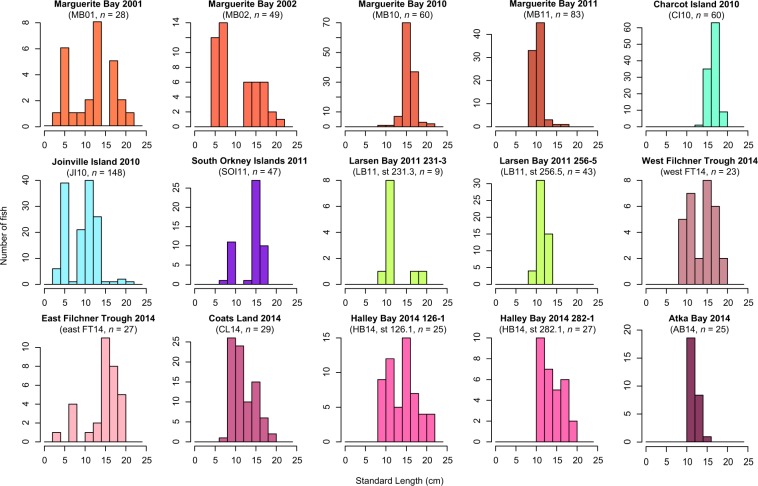
Standard length (SL) distributions of *Pleuragramma antarctica* around the Antarctic Peninsula and Weddell Sea. Sampling locations and collection years are specified above the graphs, as well as group sizes (*n*). When several collections were performed within the same year and area, sampling stations are listed after the collection year. Frequency indicated on the y-axis, SL in cm indicated on the x-axis, and the size interval is 2 cm.

Standard length (SL) distributions varied considerably between sampling locations (Fig. 4). Agostini *et al*.^19^ observed a pronounced bimodal distribution in the sample taken from Joinville Island 2010 (JI10), with modes at SL = 5 cm and SL = 10 cm. After dividing the JI10 group into small and large fish representative of more and less recent cohorts, pairwise *F*_ST_ analysis revealed that the small fish (*n* = 68) and large fish (*n* = 72) were significantly different. Seven of eight comparisons were significant between all groups and JI10 small, whereas three of eight comparisons were significant between all groups and JI10 large after SGoF + correction for multiple tests^19^ (threshold for significance with 190 comparisons *P* = 0.0453). When this analysis was extended to the sampling locations considered in the current study, this result was largely replicated: six of eight comparisons were significant between the original AP groups and JI10 small, and two of eight comparisons were significant between the original AP groups and JI10 large after SGoF + correction for multiple tests, (Table S7). However, SOI was significantly different from JI10 small (*F*_ST_ = 0.0045, *P* = 0.0265) and JI10 large (*F*_ST_ = 0.0042, *P* = 0.0312), in contrast to the original analysis. Consistent with results seen in the initial and replicated AP analysis between JI10 small and Larsen Bay 2007 (*n* = 46), JI10 small significantly differentiated after SGoF + correction for multiple tests from Larsen Bay 2011 (*n* = 52, *F*_ST_ = 0.0037, *P* = 0.0361), a comparison that was not significant when analyzing the undivided JI10 group. Similarly, the comparison between JI10 small and Ross Sea 1997 (*n* = 84) was significant after SGoF + correction for multiple tests (*F*_ST_ = 0.0044, *P* = 0.0061), despite no indication of differentiation with the undivided JI10 group. Finally, seven additional pairwise comparisons were significant after SGoF + correction for multiple tests in the JI10 cluster analysis, despite not involving the newly introduced JI10 small or large clusters (see Table S7 for details).

Spatially recurring length modes among sampling locations collected in the eastern Weddell Sea in 2014 provided evidence for episodic connectivity (Fig. 4). Thus, a separate pairwise *F*_ST_ analysis was performed including only sampling locations collected in 2014 within the eastern Weddell Sea. No significant differences were seen between any of the sampling areas, which included west Filchner Trough (*n* = 23), east Filchner Trough (*n* = 27), Coats Land (*n* = 29), Halley Bay (*n* = 52), and Atka Bay (*n* = 25) (*F*_ST_ ranged from −0.0062 to 0.0027, *P* > 0.05 for all comparisons, Table S8). With such low levels of differentiation between areas despite the diverse SL distributions, a final pairwise *F*_ST_ analysis was performed on groupings of fish from the eastern Weddell Sea that were based solely on SL range, independent of sampling location. SL among specimens from the eastern Weddell Sea ranged from 7.0–21.5 cm. First, fish were divided into six length-based groups (<10 cm, 10–12 cm, 12–14 cm, 14–16 cm, 16–18 cm, >18 cm), with group *n* ranging from 14–43 individuals. When no significant differences were found between length-based groups after SGoF + correction for multiple tests (*F*_ST_ ranged from −0.0081 to 0.01019), an exploratory analysis of various pools of the six original length-based groups was conducted. The smallest length-based pool was defined as SL ≤ 12 cm and then subsequently as SL ≤ 14 cm. The largest length-based pool was defined as SL < 16 cm and various combinations of the intermediate groups were created. Testing these length-based pools allowed for the control of the influence of group size, and none of the pairwise comparisons were significant after SGoF + correction for multiple tests (*F*_ST_ ranged from −0.0081 to 0.0103).

## Discussion

The present study tests the hypothesis of genetic panmixia^28^ in the light of the physical-biological framework provided by Ashford *et al*.^6^ for Antarctic silverfish. Evidence of high gene flow between regions connected by the Antarctic Slope Front (ASF) and associated Antarctic Slope Front Current on a circumpolar scale was accompanied by indications of reduced gene flow in regions not reached by the ASF. The Discriminant Analysis of Principle Components (DAPC), revealed evidence of nine clusters, but these were independent of geographic region. Instead, they may have arisen due to historical extinction and recolonization events predicted by Ashford *et al*.^6^, related to glacial periods that recurrently structured past populations of silverfish. Overall levels of genetic differentiation were low (maximum *F*_ST_ = 0.0153), and no significant differences were found from the Ross Sea westward to the northern Antarctic Peninsula. However, limited evidence of genetic structuring was found. We corroborated the findings from Agostini *et al*.^19^ that Charcot Island and Marguerite Bay represent a homogeneous population with significantly reduced gene flow from populations around the AP and beyond, while also discovering significant reductions in gene flow to the population at the South Orkney Islands. These results were broadly consistent with the Matschiner *et al*.^28^ prediction of gene flow facilitated by the large-scale circulation. However, the genetic structuring we found corresponded to discontinuities between major current systems along the shelf. Accordingly, we argue for a more nuanced version of the genetic panmixia hypothesis that incorporates physical-biological interactions over silverfish life history.

### Along-shelf gene flow and connectivity

Similarity between samples from the Ross Sea, eastern Weddell Sea, Larsen Bay and Joinville Island indicated gene flow along the shelf corresponding to the ASF. Pairwise *F*_ST_ analysis provided evidence for six possible silverfish populations among the geographic regions analyzed. Moreover, the Ross Sea did not differ from the Weddell Sea, Larsen Bay or Joinville Island. Joinville Island and the South Shetland Islands formed a cluster, which did not differ from the others, suggesting gene flow along the ASF into the Bransfield Strait. Notably, comparisons made by Agostini *et al*.^19^ showed evidence of differentiation between Larsen Bay and Joinville Island, whereas in the present analysis, not a single comparison among sampling locations from Joinville (JI07, JI10, and JI12) and Larsen Bay (LB07 and LB11) revealed significant differentiation. This is consistent with the predictions made in La Mesa *et al*.^26^ of connectivity between Larsen Bay and the Bransfield Strait. Thus, the inclusion of the additional samples from Larsen Bay (LB11) clarified connectivity along the ASF between the western Weddell Sea and Bransfield Strait.

In contrast, regions not connected by the ASF showed evidence of reduced gene flow. Along the western Antarctic Peninsula, where westward flow inshore along the AACC contrasts with eastward flow along the slope in the southern ACC, Charcot Island (CI) and Marguerite Bay (MB) represented one cluster, which differentiated genetically from all other geographic regions. Discontinuity in silverfish distribution between the northern and southern regions of the western Antarctic Peninsula was first discussed in Agostini *et al*.^19^ based in part on the decrease of silverfish in Adélie penguin diets at Palmer Station^42^. The loss of intermediate populations that may have previously facilitated connectivity between the northern and southern extents of the western Antarctic Peninsula was further supported by the total absence of silverfish in sampling surveys at Anvers and Renaud Islands and the Croker Passage midway up the western Antarctic Peninsula in 2010 (see Fig. 1, WAP for place names) despite having previously been the dominant fish species in these areas since the 1980s^14^. In addition, the South Orkney Islands differentiated from all other regions except for the eastern Weddell Sea. Differentiation between years within certain sampling locations echoed evidence for chaotic genetic patchiness seen in the Ross Sea by Zane *et al*.^36^ and in the Antarctic Peninsula by Agostini *et al*.^19^. This was further indicated by the variability in SL distributions between years in particular sampling locations, providing evidence for variation in recruitment and dispersal of different cohorts^37^.

The clearest example of interannual variability was seen in the western Antarctic Peninsula (WAP). Specimens from MB01 and MB02 exhibited a bimodal distribution of SL representative of more and less recent cohorts. In contrast, specimens from MB10 and CI10 exhibited a much tighter unimodal distribution around SL = 15 cm, as well as MB11, which had a similarly tight unimodal distribution around SL = 10 cm (Fig. 4). This disparity between cohorts was reflected in patterns of genetic differentiation within the WAP area between years, with MB01 and MB02 differentiating from two of the nineteen other sampling locations, while MB11 differed from six of the nineteen other sampling locations (Table 3). However, position with respect to local hydrographic features must also be considered. Despite being collected only a year before the strongly differentiated MB11 sample, MB10 shares the weak differentiation pattern of nearby MB01 and MB02, which were all collected in the Labeuf Fjord closer to the coast (Fig. 1, WAP, Table 3). This greater connectivity with populations outside of the WAP is consistent with gene flow down the Antarctic Peninsula promoted by the AACC^19^. Furthermore, the position of MB11 along the trough inflow in Marguerite Trough (Fig. 1, WAP) raises the possibility that the sample consisted of fish resulting from connectivity along the slope, where entrainment in the southern ACC potentially transports fish from the Bellingshausen and Amundsen Seas^23^. The influx of migrants from populations further upstream may contribute to the stronger pattern of differentiation found between MB11 and sampling locations beyond the Antarctic Peninsula.

Differences between the previously published Antarctic Peninsula study^19^ and the current results are likely attributable to changes in significance thresholds resulting from the increased number of comparisons in the present study, as well as the use of 15 instead of 16 loci due to the removal of one problematic locus. In the original Antarctic Peninsula analysis, Agostini *et al*.^19^ found 9 out of 36 significant comparisons around the Antarctic Peninsula, while in the current analysis, only four out of the thirty-six Antarctic Peninsula comparisons were significant. This was mainly due to weaker differentiation between WAP locations and JI10 and JI12. Discrepancies in patterns of differentiation between the current study and the original investigation of population connectivity in Zane *et al*.^36^ can be attributed to their use of mtDNA as opposed to microsatellite markers to resolve patterns of genetic heterogeneity^40^, in addition to differences in sample size (Table 3). Given the small differences geographically, variation associated with reproduction, differential survival, and recruitment can lead to changes in resolution in subsequent investigations^6^. These results highlight the importance of continued, targeted sampling that incorporates hydrography in order to monitor changes in population structure over time. The integration of multidisciplinary techniques, notably circulation and movement modelling^43^, otolith chemistry^44^, and genomics^45^, will also help in resolving population structure and connectivity over the life history.

### Population structure and life history connectivity

The silverfish physical-biological population hypothesis^6^ provides a mechanistic explanation consistent with the patterns of gene flow and genetic structuring we observed. When fish in outflows from local trough systems, for instance in the Ross Sea^20^ and eastern Weddell Sea^22^, reach the shelf break, entrainment in the ASF and westward transport has the potential to introduce fish into trough systems downstream. Samples from Terra Nova Bay in the Ross Sea collected in 1996 and 1997 were genetically very similar to those collected in the Weddell Sea and Antarctic Peninsula, only differentiating from samples taken along the western Antarctic Peninsula and the South Orkney Islands. This high level of connectivity between regions on opposite sides of the continent may be attributed to two main factors. The first is related to the extent and importance of the Terra Nova Bay silverfish nursery, where silverfish comprise 98% of the ichthyoplankton^4,13^. The second is related to potential silverfish populations along East Antarctica. Despite the extensive geographic purview of this study, significant gaps remain for this circumpolar species, especially along East Antarctica. Substantial distributions of silverfish have been found across the continental shelf along East Antarctica^3^, as well as in association with the Ninnis and Adélie Troughs off Wilkes Land^46^ (Fig. S4, East Antarctica). Other trough systems along East Antarctica in Lützow-Holm Bay, Iceberg Alley, Nielsen Basin, and Prydz Bay may also provide suitable habitats for coherent silverfish populations^6^ (Fig. S4, East Antarctica). The existence of a network of local populations of silverfish associated with trough systems along East Antarctica provides a mechanism for the exchange of individuals via the westward-flowing ASF. Such a mechanism would explain the high levels of gene flow observed between sampling locations as geographically separated as Terra Nova Bay and Joinville Island.

Moreover, our results lend further support for connectivity beyond Joinville Island into the region around the Bransfield Strait. Fish at Joinville could be transported there by either: (1) the AACC, which could transport fish from Larsen Bay^26,47^; or (2) the ASF, which could transport enough individuals from the eastern Weddell Sea and beyond to render the Joinville population genetically indistinguishable from upstream populations^6,9,48^. Although the path of the AACC south of Joinville Island is undefined, buoyancy forcing is likely to generate connectivity that is shallow and close to the coast^7^. This could result in larval transport north from Larsen Bay which would explain the differentiation between size modes in the Antarctic Sound (Fig. 1., NAP).

The results of the current study raise the possibility of a coherent silverfish population at the South Orkney Islands. Surveys carried out between 1967–1981 revealed the presence of silverfish larvae at the South Orkney Islands^38^, consistent with a local breeding population. Furthermore, a recent bathymetric analysis of the South Orkney Islands microcontinent found seven trough systems on the northern and southern sides of the islands^49^. Features supportive of a locally breeding silverfish population were found in Signy Trough between Coronation and Signy Island^49^, including active glaciers at the trough head, thought to be necessary for early-life stages^6^ (Fig. 1, SOI). Evidence from otolith chemistry analyses has revealed that fish from the northern Antarctic Peninsula significantly differed from those at the South Orkney Islands, indicating that fish in these areas were sourced from different spawning grounds^50^. This is supported by our findings of genetic differentiation between the northern Antarctic Peninsula and South Orkney Islands populations.

However, the lack of differentiation between specimens from the South Orkney Islands and specimens from the eastern Weddell Sea means connectivity between these two regions cannot be discounted based on these data. The existence of migrants or non-breeding vagrants at the South Orkney Islands would be predicated by a clear and consistent transport system from the eastern Weddell Sea to the South Orkney Islands microcontinent. Although fish carried to the shelf slope along trough outflows in the eastern Weddell Sea can come into contact with the ASF^6^, subsequent transport to the South Orkney Islands microcontinent remains unclear. In addition to the distance along the ASF, fish are potentially exposed to inflows at the mouths of the Hughes, Ronne (Fig. 1, WS), Jason, and Robertson (Fig. 1, LB) Troughs that can draw them away from the slope towards the inner shelf. Continued entrainment in the ASF north of Larsen Bay carries fish around Joinville Island, where the AACC merges with the ASF. Subsequently, the currents separate again, the AACC forming the southern branch of the Bransfield Gyre^10^ (Fig. 1, NAP) and the ASF moving north along the South Scotia Ridge^9,47,51^. However, upon entering the Hesperides Trough, increased influence by Weddell-Scotia Confluence waters combined with local bathymetric complexity impede continued identification of the ASF^51^. Thus, the ASF does not provide a direct pathway to the South Orkney Islands. Alternatively, the Weddell Front, which forms east of the Joinville Ridge and rounds the Powell Basin to reach the South Orkney Islands microcontinent, may represent a path from the Weddell Sea to the South Orkney Islands. Future studies will require an interdisciplinary approach to assess the impact of episodic connectivity on genetic similarity between the South Orkney Islands and the eastern Weddell Sea. The hydrography between these areas needs to be elaborated in order to determine the eventual fate of the ASF. Otolith chemistry analyses^52^ and dispersal modelling^53^ of fish from the South Orkney Islands and the eastern Weddell Sea will be able to clarify how much the genetic similarity between these regions is due to episodic connectivity into the local population.

Hydrography may also explain the chaotic genetic patchiness observed in the differentiation patterns between the two Larsen Bay samples and the eastern Weddell Sea and South Orkney Islands (Table 3). It is important to note that the LB07 and LB11 samples were collected from different parts of Larsen Bay (Fig. 1, LB). LB07 was collected in the Larsen B embayment off Scar Inlet, near one of the heads of the Larsen Inlet Trough. The LB11 samples were collected in the Larsen A embayment, just north of the Seal Nunataks Ice Shelf, at the head of the Robertson Trough. Since the disintegration of the Larsen A and B shelves, runoff into the Larsen embayments has been derived from smaller glaciers along the peninsula that contribute to disparate patterns of freshwater influx between the Larsen A and B areas^54,55^. Differences in seasonal melting patterns between the two areas are likely to directly impact coastal buoyancy and connectivity between trough systems in Larsen Bay. Considering the evidence of gene flow between the eastern Weddell Sea and both the Larsen Bay and South Orkney Islands, the lack of differentiation between LB11 and SOI11 may be due to changes in the influx of eastern Weddell Sea migrants into these areas. This is in contrast to the hypothesis that the observed gene flow results from the direct exchange of individuals between Larsen Bay and the South Orkney Islands. Changes in recruitment may occur in tandem with the influx of eastern Weddell Sea migrants into the respective Larsen A and Larsen B embayments. This would result in the variations of gene flow observed between the eastern Weddell Sea, South Orkney Islands, and the LB07 and LB11 sampling locations.

The pattern of homogeneity observed among fish across all sampling locations within the eastern Weddell Sea fails to explain the variation in SL distributions. This suggests population mixing between local and migrant fish, seen from Atka Bay in the northeast, to Halley Bay and Coats Land, and finally to the eastern and western sides of the Filchner Trough in the southeast. Aspects of local hydrography help to explain these observations. In the southeast Weddell Sea, the AACC and the ASF are functionally equivalent given the narrowness of the continental shelf in this area^9^. Seasonal and interannual variability in the AACC in the southeast Weddell Sea^56^ may promote and restrict connectivity along the shelf from Atka Bay to Filchner Trough. Fish that become entrained in the ASF upstream are introduced to the Weddell Sea east of Atka Bay, as it becomes the southern limb of the Weddell Gyre^8,57^. Having formed in the Amundsen Sea, the ASF has the potential to connect local trough systems in the Ross Sea and East Antarctica^6,8^. While it remains a more consistent feature than the AACC, the position and strength of the ASF is nevertheless sensitive to wind stress forcing on seasonal and interannual time scales in the northwest Weddell Sea^51,58,59^. Such variations in the intensity of the ASF could explain fluctuations in the influx of migrants into eastern Weddell Sea populations and provide a general mechanism for the chaotic genetic patchiness observed among silverfish populations.

While the present dataset is unique in both its temporal and spatial extents, as well as in its inclusion of life history data, it is important to keep in mind its limitations and what types of analyses and sampling could fill the present gaps. Variation in collection gear by research vessel between sampling locations (Table 1) may have introduced bias into the allele frequencies measured between location. However, the high levels of homogeneity found between locations sampled by different research vessels, coincident with differences between locations sampled by the same research vessels, imply that any gear biases did not have a consequential impact on the present results. Subsequent studies analyzing otolith nucleus chemistry in silverfish along the eastern Weddell Sea will allow us to distinguish between areas more heavily composed of locally spawned fish and migrants. Furthermore, enhanced oceanographic data would improve dispersal modelling, allowing for a more targeted approach in defining connectivity hypotheses. Contiguous sampling from regions that could contribute migrating fish to the ACC and the ASF are needed in order to better define in which ways the various current systems influence eastern Weddell Sea population mixing.

Nevertheless our results show no basis for rejecting along-shelf connectivity predicted by Ashford *et al*.^6^, and are consistent with the cross-shelf component of their hypothesis, examined earlier by Brooks *et al*.^20^ in the Drygalski Trough. Moreover, the implications of observed structure and gene flow in Antarctic silverfish is seen in other Antarctic species. Krill (*Euphasia superba*), a keystone species in waters beyond the continental shelf^60^, exhibit a similar lack of population structure, as shown in studies using mtDNA and microsatellites^61^, as well as restriction-site-associated DNA sequencing^62^. Such panmixia observed in Antarctic krill was explained by very large population sizes and ongoing gene flow^61,62^. However, it was stressed that genetically indistinguishable populations do not imply ecologically independent populations^61,63^. Evidence for a recent population expansion observed in analysis of mtDNA in Bortolotto *et al*.^61^ emphasized the differential impact of glaciations on the population structure of pelagic versus benthic Antarctic species. Thus, impacts of climate change may result in range restrictions that will have the opposite effect of glacial expansions on pelagic species, cutting off intermediate populations which can result in cascading effects on the connectivity of ecologically interdependent populations^64,65^.

Antarctic silverfish are a pillar of the Southern Ocean ecosystem^2^. Understanding their population structure and connectivity in the context of their life history and the impact of hydrography is critical to assessments of Southern Ocean ecosystem health.

## Materials and Methods

### Sample collection and DNA extraction

A total of 1067 individuals from 19 sampling locations collected by multiple institutions between 1989 and 2014 were included in the analysis (Fig. 1, Table 1). Of these, 249 individuals collected between 1989 and 1997 from the Ross Sea (RS96, RS97), Halley Bay (HB89, HB91), and South Shetland Islands (SSI96) were included from a previous study on population structure using mitochondrial DNA^36^. A further 562 individuals collected between 2001 and 2012 from Larsen Bay (LB07), Charcot Island (CI10), Marguerite Bay (MB01, MB02, MB10, MB11), and Joinville Island (JI07, JI10, JI12) were included from a previous connectivity study focused on the Antarctic Peninsula^19^. The remaining 256 individuals collected in 2011 and 2014 from the South Orkney Islands (SOI11), Larsen Bay (LB11), Filchner Trough (FT14), Atka Bay (AB14), and Halley Bay (HB14) have not previously been examined. Table 1 outlines sampling details, including research vessel information and collection gear. All samples, including those obtained in 2011 and 2014 that had not been previously examined, were collected during authorized scientific cruises carried out by nations legally committed to the Convention for the Conservation of Antarctic Marine Living Resources^66^. No manipulations were carried out on live samples.

Standard length (SL) data were available from all individuals except from those collected from RS96, RS97, and HB91. SL data from HB89 and SSI96 represent the length distribution of fish collected at the sampling stations, but lack identification numbers to correspond them to the tissue samples collected for genetic analysis. The distribution of SL was used to identify modes for consideration in the eventual genetics analysis.

Muscle tissue or fin clips were preserved in 95–100% ethanol at the time of sampling. Genomic DNA was extracted from specimens using a standard salting-out procedure^67^. Genomic DNA was extracted from all previously unexamined samples, as well as from tissue samples remaining from individuals on which the mitochondrial DNA analysis was performed in Zane *et al*.^36^. Concentration and quality of the extracted DNA (260/280 nm and 260/230 nm) was checked using a NanoDrop UV–Vis spectrophotometer (Thermo Scientific) prior to PCR amplification. All extracted DNA was of high enough quality to use in subsequent PCR reactions.

### DNA amplification and microsatellite genotyping

Individuals were genotyped using 16 published EST-linked microsatellites developed in *Chionodroco hamatus*^68,69^, that had been previously shown to cross-amplify successfully in *P. antarctica*^19^. The 16 loci were amplified in two multiplex PCR reactions (see Table S2 for primer sequences and final conditions for all loci). Multiplex PCR reaction volume was 10 µL, containing 1x QIAGEN Multiplex PCR Master mix (HotStartTaq DNA Polymerase, Multiplex PCR Buffer, dNTP Mix; QIAGEN, Hilden, Germany), 0.2 µM primer mix and 100 ng of genomic DNA. The PCR amplification profile for all loci consisted of: (1) an initial activation step of 15 min at 95 °C; (2) 30 cycles of denaturation at 94 °C for 30 s, annealing at 57 °C for 90 s, and extension at 72 °C for 60 s; and (3) a final extension of 30 min at 60 °C.

PCR products were prepared for microsatellite genotyping and sent to an external service (BMR Genomics, http://www.bmr-genomics.com/), where they were sequenced using an ABI 3730xl automated sequencer (LIZ 500 as internal size ladder, Applied Biosystems, Waltham, MA, USA). Microsatellites were analyzed using GeneMarker ver. 2.6 (SoftGenetics). Genotypes of individuals included in the microsatellite analysis published by Agostini *et al*.^19^ were integrated into this analysis. To ensure that the datasets were comparable, two individual samples from Agostini *et al*.^19^ were processed alongside samples from the present study as positive controls during each amplification and sequencing run. This allowed the comparison of the raw microsatellite data between the previous and current study and confirmed that there was no change in microsatellite sizing. Binning was automated with FlexiBin 2^70^ and refined by eye to assure accuracy with the corresponding binnings established in Agostini *et al*.^19^.

### Data analysis

A suite of programs was used to assess population differentiation and genetic structuring based on the microsatellite genotypes. Input files were created in the appropriate software-specific formats using CREATE ver 1.37^71^. Linkage Disequilibrium (LD) probability was tested with GENEPOP online^72^. Deviations from Hardy-Weinberg equilibrium were calculated with the R package diveRsity ver. 1.9.89^73^. The software Micro-Checker 2.2.3 was used to detect the presence of null alleles and scoring errors due to large allele drop-out and stuttering^74^. Significance levels for multiple comparisons were adjusted using the program SGoF+ ^75^.

Detection of loci under selection was carried out using the methods implemented in LOSITAN ver. 1.0.0^76^ and ARLEQUIN 3.5.1.3^77^. Both software generate coalescent simulations based on observed data and a chosen demographic model of migration to create the expected distribution of *F*_ST_ versus *H*_E_ with neutral markers. Outlier loci are detected based on the presence of significantly higher or lower *F*_ST_ values compared to neutral expectations. In LOSITAN, 1,000,000 simulations were run under neutral mean *F*_ST_, simulating 19 groups (for each sampling location), confidence interval of 0.95% and a false discovery rate of 0.01 assuming a Stepwise Mutation Model (SMM)^19^. In ARLEQUIN, genetic structure was assessed by running 50,000 simulations, simulating 19 groups (for each sampling location) and 6 groups (corresponding to the number of geographic regions), and assuming 100 demes per group under the hierarchical island model^78^.

Within-sampling location genetic variability was assessed by calculating observed (*H*_*O*_) and expected (*H*_*e*_) heterozygosity, number of alleles (*N*_*A*_), and allelic richness (*A*_*R*_) using the R package diveRsity ver. 1.9.89^73^. Genetic variability was compared between sampling locations using a one-way ANOVA performed in R^79^.

The extent to which sampling locations could be clustered into the same group was assessed using various methods. Discriminant analysis of principal components (DAPC), a multivariate method in which fish are assigned to known groups, and Principle Coordinate Analysis (PCoA), a multidimensional scaling method in which the matrix of dissimilarities was built from *F*_ST_ comparisons, were carried out using the R package ADEGENET 2.0.1^80^ and GenAlEx 6.501^81,82^, respectively, to provide a visual assessment of differentiation between sampling locations. Estimation of the number of groups into which genotypes from the various sampling locations cluster, ignoring *a priori* information about provenance, was tested using FLOCK 3.1^83^. FLOCK employs a non Markov Chain Monte Carlo (MCMC) algorithm that uses an iterative method to divide genotypes into *k* clusters. This method has been shown to allocate clusters more accurately than common Bayesian methods when population structure is weak^84^.

The power of microsatellite loci to detect differences in the sample sizes analyzed was assessed applying Fisher’s and Chi-square tests in POWSIM ver 4.1^85^. Observed sample sizes and allele frequencies taken from the 19 sampling locations were used to gauge the statistical power of the 15 microsatellites to detect *F*_ST_ values ≤ 0.01 using 1000 replicates. In accordance with the POWSIM manual, several combinations of N_e_ (1000–10,000) and generations (t = 2–201) were tested to account for variability in the estimation of N_e_.

Population differentiation was determined by calculating pairwise genetic distances represented by *F*_ST_^86^ between sampling locations as well as between SL-defined groups. Hierarchical analysis of molecular variance (AMOVA) was also used to assess population differentiation. Pairwise genetic differences and AMOVA were calculated in ARLEQUIN 3.5.1.3^77^. Significant differentiation was determined with 10,000 permutation tests. The significance threshold was adjusted according to SGoF + correction for multiple tests^75^.

## Electronic supplementary material

Supplementary Information

## Data Availability

The datasets generated and analyzed during the current study are available from the corresponding author on request.
