# Supplementary material for: Along-shelf connectivity and circumpolar gene flow in Antarctic silverfish (Pleuragramma antarctica)
Source: Sci Rep. 2018 Dec 14;8:17856. doi: 10.1038/s41598-018-36030-x (PMC6294782; doi:10.1038/s41598-018-36030-x)
Supplement: Supplementary file 1 — Supplementary Information [file 41598_2018_36030_MOESM1_ESM.pdf]

Supplementary Information Along-shelf connectivity and circumpolar gene flow in Antarctic silverfish (*Pleuragramma antarctica*)

Jilda Alicia Caccavo[1*], Chiara Papetti[1,2], Maj Wetjen[3], Rainer Knust[4], Julian R. Ashford[5], Lorenzo Zane[1,2]

[1] Department of Biology, University of Padua, Via G. Colombo 3, Padua 35121, Italy.

[2] Consorzio Nazionale Interuniversitario per le Scienze del Mare (CoNISMa), Piazzale Flaminio 9, Rome 00196, Italy.

[3] Institute for Environmental Sciences, University of Koblenz-Landau, Fortstraße 7, Landau 76829, Germany.

[4] Helmholtz Center for Polar and Marine Research, Alfred Wegener Institute, Am Alten Hafen 26, Bremerhaven 27568, Germany.

[5] Department of Ocean, Earth and Atmospheric Sciences, Center for Quantitative Fisheries Ecology, Old Dominion University, 800 West 46th Street, Norfolk, VA 23508, United States.

Corresponding author

* Jilda Alicia Caccavo, ergo@jildacaccavo.com, ORCID: 0000-0002-8172-7855

Table S1 Genetic variability of *Pleuragramma antarctica* for each locus across all nineteen sampling locations. Sample size (*n*), number of alleles (N_A), % of total observed alleles per locus per sampling location, allelic richness based on a minimum sample size of 14 individuals (A_R), observed heterozygosity (H_O), unbiased heterozygosity (H_E) and probability of deviation from Hardy-Weinberg equilibrium (*pHWE*) are shown. Values in bold indicate significant HWE deviations after correction for multiple tests as implemented in SGoF+[1] (threshold for significance with 304 comparisons P = 0.008). Sampling location acronyms are as in Table 1.

	**CI10**							**MB01**							**MB02**						
**Locus**	***n***	N_A	**%**	A_R	H_O	H_E	***pHWE***	***n***	N_A	**%**	A_R	H_O	H_E	***pHWE***	***n***	N_A	**%**	A_R	H_O	H_E	***pHWE***
**Ch126**	60	3	42.86	2.21	0.45	0.51	0.300	28	3	42.86	2.38	0.46	0.51	0.711	49	4	57.14	3.00	0.59	0.55	0.763
**Ch520**	60	4	50.00	3.09	0.33	0.38	0.488	28	4	50.00	3.38	0.39	0.51	0.020	49	5	62.50	3.70	0.47	0.48	0.754
**Ch623**	60	23	69.70	14.35	0.92	0.94	**<0.001**	28	20	60.61	12.75	0.96	0.92	0.219	49	20	60.61	12.82	0.90	0.92	0.975
**Ch10105**	60	6	66.67	3.54	0.30	0.31	0.146	28	3	33.33	1.86	0.07	0.07	1.000	49	5	55.56	2.89	0.24	0.24	0.182
**Ch10857**	60	9	52.94	6.34	0.68	0.69	0.903	28	9	52.94	6.03	0.54	0.66	0.091	49	7	41.18	5.59	0.57	0.67	0.702
**Ch13222**	60	3	50.00	2.26	0.17	0.16	1.000	28	2	33.33	1.94	0.18	0.16	1.000	49	4	66.67	1.75	0.06	0.06	1.000
**Ch18085**	60	10	71.43	7.29	0.83	0.83	0.852	28	9	64.29	6.98	0.86	0.82	0.790	49	9	64.29	7.02	0.86	0.83	0.998
**Ch2931**	60	10	58.82	6.96	0.73	0.83	0.891	28	10	58.82	7.55	0.86	0.82	0.165	49	11	64.71	7.09	0.84	0.82	0.370
**Ch4796**	60	13	72.22	9.07	0.75	0.87	**0.002**	28	12	66.67	9.71	0.96	0.88	0.985	49	14	77.78	10.09	0.94	0.89	0.793
**Ch10441**	60	9	39.13	5.36	0.65	0.65	0.643	28	10	43.48	7.17	0.86	0.76	0.535	49	9	39.13	6.00	0.61	0.68	0.524
**Ch11230**	60	7	50.00	4.90	0.77	0.72	0.744	28	5	35.71	4.69	0.71	0.71	0.996	49	7	50.00	5.00	0.78	0.72	1.000
**Ch11483**	60	10	66.67	5.43	0.47	0.56	0.675	28	9	60.00	6.26	0.57	0.64	0.409	49	9	60.00	4.67	0.45	0.46	0.096
**Ch17977**	60	8	53.33	5.08	0.58	0.63	0.722	28	4	26.67	3.60	0.57	0.56	0.955	49	7	46.67	4.60	0.65	0.62	0.509
**Ch19846**	60	8	66.67	6.26	0.70	0.78	0.690	28	8	66.67	6.56	0.86	0.78	0.996	49	8	66.67	6.52	0.86	0.80	0.941
**Ch24332**	60	6	60.00	4.02	0.60	0.62	0.096	28	5	50.00	3.92	0.57	0.60	0.320	49	5	50.00	3.70	0.73	0.62	0.184
**Ch25478**	60	4	25.00	2.96	0.57	0.56	0.422	28	4	25.00	3.04	0.46	0.56	0.036	49	6	37.50	3.69	0.61	0.63	0.341

	MB10							MB11							JI07						
**Locus**	***n***	N_A	**%**	A_R	H_O	H_E	***pHWE***	***n***	N_A	**%**	A_R	H_O	H_E	***pHWE***	***n***	N_A	**%**	A_R	H_O	H_E	***pHWE***
**Ch126**	60	4	57.14	2.40	0.43	0.52	0.205	83	3	42.86	2.77	0.58	0.55	0.661	34	5	71.43	3.22	0.56	0.54	1.000
**Ch520**	60	5	62.50	4.07	0.62	0.56	0.855	83	5	62.50	3.58	0.45	0.40	0.712	34	3	37.50	2.95	0.50	0.41	0.362
**Ch623**	60	24	72.73	13.71	0.93	0.93	0.038	83	25	75.76	13.58	0.89	0.93	0.012	34	22	66.67	13.01	0.97	0.92	0.106
**Ch10105**	60	4	44.44	2.88	0.22	0.21	0.594	83	5	55.56	3.16	0.25	0.23	0.680	34	4	44.44	2.99	0.24	0.22	1.000
**Ch10857**	60	11	64.71	6.36	0.75	0.73	0.121	83	10	58.82	6.32	0.73	0.71	0.937	34	7	41.18	5.29	0.76	0.74	0.716
**Ch13222**	60	4	66.67	2.40	0.25	0.22	1.000	83	3	50.00	1.94	0.10	0.10	**<0.001**	34	3	50.00	2.31	0.21	0.23	1.000
**Ch18085**	60	10	71.43	7.21	0.83	0.83	0.171	83	11	78.57	7.05	0.87	0.82	0.959	34	10	71.43	7.47	0.76	0.82	0.715
**Ch2931**	60	10	58.82	7.52	0.83	0.81	0.998	83	10	58.82	6.91	0.73	0.81	0.279	34	10	58.82	7.61	0.71	0.84	0.333
**Ch4796**	60	13	72.22	9.37	0.82	0.88	0.996	83	14	77.78	10.72	0.93	0.90	1.000	34	11	61.11	9.11	1.00	0.88	1.000
**Ch10441**	60	11	47.83	7.20	0.73	0.71	0.436	83	12	52.17	6.09	0.69	0.68	0.989	34	9	39.13	5.85	0.74	0.66	0.289
**Ch11230**	60	8	57.14	5.68	0.68	0.76	0.986	83	6	42.86	4.64	0.80	0.73	0.603	34	7	50.00	5.71	0.88	0.76	0.547
**Ch11483**	60	8	53.33	5.20	0.65	0.57	0.597	83	8	53.33	4.26	0.55	0.56	0.461	34	5	33.33	3.77	0.59	0.48	0.606
**Ch17977**	60	9	60.00	5.13	0.62	0.66	**<0.001**	83	10	66.67	4.85	0.58	0.58	0.013	34	7	46.67	5.73	0.68	0.69	0.828
**Ch19846**	60	8	66.67	6.45	0.87	0.80	0.216	83	8	66.67	6.04	0.71	0.78	0.618	34	7	58.33	5.72	0.76	0.77	0.385
**Ch24332**	60	6	60.00	4.64	0.60	0.65	0.934	83	7	70.00	4.55	0.69	0.63	0.914	34	4	40.00	3.79	0.62	0.64	0.800
**Ch25478**	60	6	37.50	4.43	0.68	0.64	0.018	83	8	50.00	4.45	0.64	0.63	0.686	34	7	43.75	4.12	0.47	0.61	0.019

	JI10							JI12							SSI96						
**Locus**	***n***	N_A	**%**	A_R	H_O	H_E	***pHWE***	***n***	N_A	**%**	A_R	H_O	H_E	***pHWE***	***n***	N_A	**%**	A_R	H_O	H_E	***pHWE***
**Ch126**	148	6	85.71	3.08	0.63	0.55	0.384	54	5	71.43	3.00	0.50	0.54	1.000	14	2	28.57	2.00	0.57	0.49	0.620
**Ch520**	148	8	100.00	4.15	0.57	0.58	0.713	54	6	75.00	3.87	0.44	0.51	0.210	14	5	62.50	4.15	0.50	0.42	1.000
**Ch623**	148	28	84.85	13.77	0.91	0.94	0.574	54	20	60.61	12.56	0.80	0.92	0.092	14	15	45.45	11.59	0.86	0.91	**<0.001**
**Ch10105**	148	6	66.67	3.72	0.32	0.37	0.074	54	5	55.56	3.35	0.20	0.25	0.359	14	3	33.33	2.66	0.36	0.30	1.000
**Ch10857**	148	12	70.59	6.60	0.70	0.74	0.858	54	11	64.71	6.33	0.74	0.71	0.090	14	5	29.41	4.59	0.64	0.69	0.170
**Ch13222**	148	4	66.67	2.26	0.16	0.17	0.718	54	3	50.00	2.10	0.15	0.14	1.000	14	3	50.00	2.29	0.14	0.14	1.000
**Ch18085**	148	12	85.71	7.69	0.84	0.85	0.931	54	8	57.14	6.78	0.85	0.81	0.507	14	6	42.86	5.74	0.71	0.80	0.879
**Ch2931**	148	10	58.82	7.02	0.78	0.81	0.297	54	8	47.06	6.19	0.67	0.82	0.999	14	7	41.18	6.39	0.79	0.82	0.475
**Ch4796**	148	14	77.78	9.56	0.82	0.89	0.491	54	12	66.67	8.86	0.98	0.87	0.991	14	11	61.11	8.89	0.93	0.86	0.009
**Ch10441**	148	17	73.91	6.54	0.67	0.70	**<0.001**	54	7	30.43	5.13	0.67	0.66	0.910	14	6	26.09	5.48	0.64	0.72	0.136
**Ch11230**	148	9	64.29	5.23	0.74	0.74	0.860	54	7	50.00	5.18	0.69	0.70	0.222	14	5	35.71	4.59	0.57	0.73	0.842
**Ch11483**	148	12	80.00	5.06	0.55	0.58	**<0.001**	54	8	53.33	4.43	0.54	0.57	0.739	14	5	33.33	3.80	0.29	0.37	0.082
**Ch17977**	148	9	60.00	5.20	0.60	0.63	0.035	54	8	53.33	5.52	0.63	0.63	0.308	14	4	26.67	3.53	0.64	0.56	0.663
**Ch19846**	148	10	83.33	6.36	0.80	0.79	0.250	54	8	66.67	6.50	0.78	0.79	0.386	14	8	66.67	7.07	0.86	0.83	0.811
**Ch24332**	148	8	80.00	4.87	0.62	0.65	0.842	54	6	60.00	3.80	0.57	0.59	0.013	14	5	50.00	3.97	0.71	0.62	**0.006**
**Ch25478**	148	9	56.25	4.45	0.67	0.63	0.267	54	5	31.25	3.39	0.56	0.59	0.023	14	3	18.75	2.65	0.50	0.53	0.594

Locus	SOI11							LB07							LB11						
	n	N_A	%	A_R	H_O	H_E	*pHWE*	*n*	N_A	%	A_R	H_O	H_E	*pHWE*	*n*	N_A	%	A_R	H_O	H_E	*pHWE*
**Ch126**	47	3	42.86	2.24	0.40	0.50	0.140	46	3	42.86	2.72	0.65	0.49	0.152	52	3	42.86	2.57	0.60	0.52	0.879
**Ch520**	47	6	75.00	4.54	0.51	0.60	0.108	46	5	62.50	3.96	0.50	0.60	0.328	52	7	87.50	4.52	0.58	0.57	0.948
**Ch623**	47	18	54.55	12.21	0.91	0.92	1.000	46	20	60.61	12.82	0.87	0.93	0.997	52	21	63.64	13.57	0.88	0.93	1.000
**Ch10105**	47	6	66.67	3.93	0.36	0.35	0.329	46	4	44.44	2.66	0.17	0.18	**0.001**	52	5	55.56	2.88	0.23	0.21	1.000
**Ch10857**	47	9	52.94	6.25	0.72	0.66	0.938	46	9	52.94	5.74	0.54	0.63	0.816	52	10	58.82	6.23	0.73	0.74	0.713
**Ch13222**	47	3	50.00	2.24	0.19	0.21	1.000	46	3	50.00	1.86	0.04	0.12	**0.001**	52	2	33.33	1.91	0.15	0.14	1.000
**Ch18085**	47	8	57.14	6.59	0.91	0.80	0.997	46	8	57.14	6.69	0.83	0.82	0.861	52	9	64.29	7.20	0.88	0.82	0.869
**Ch2931**	47	9	52.94	6.75	0.70	0.82	0.444	46	11	64.71	7.75	0.78	0.83	0.939	52	9	52.94	6.96	0.77	0.83	0.172
**Ch4796**	47	12	66.67	8.74	0.79	0.87	0.902	46	10	55.56	7.88	0.91	0.86	0.921	52	12	66.67	9.13	0.92	0.88	1.000
**Ch10441**	47	7	30.43	4.88	0.49	0.56	0.111	46	11	47.83	6.74	0.70	0.70	0.142	52	10	43.48	6.07	0.58	0.63	0.046
**Ch11230**	47	6	42.86	4.79	0.64	0.68	0.847	46	6	42.86	5.12	0.70	0.74	0.781	52	7	50.00	5.19	0.73	0.72	0.105
**Ch11483**	47	7	46.67	4.85	0.60	0.55	0.764	46	7	46.67	4.63	0.61	0.55	0.597	52	7	46.67	4.49	0.48	0.50	**0.001**
**Ch17977**	47	8	53.33	5.58	0.60	0.59	0.728	46	6	40.00	4.38	0.54	0.57	0.982	52	8	53.33	4.82	0.67	0.64	0.996
**Ch19846**	47	7	58.33	5.79	0.66	0.73	0.744	46	9	75.00	6.46	0.83	0.78	0.587	52	10	83.33	7.11	0.88	0.82	0.852
**Ch24332**	47	8	80.00	4.81	0.64	0.67	0.757	46	6	60.00	4.35	0.57	0.65	0.579	52	8	80.00	4.97	0.60	0.69	0.455
**Ch25478**	47	8	50.00	4.50	0.55	0.64	**<0.001**	46	5	31.25	3.79	0.67	0.59	0.784	52	7	43.75	3.82	0.50	0.59	**0.005**

	FT14							HB89							HB91						
**Locus**	***n***	N_A	**%**	A_R	H_O	H_E	***pHWE***	***n***	N_A	**%**	A_R	H_O	H_E	***pHWE***	***n***	N_A	**%**	A_R	H_O	H_E	***pHWE***
**Ch126**	50	4	57.14	3.07	0.50	0.56	1.000	19	2	28.57	2.00	0.53	0.49	1.000	41	3	42.86	2.49	0.51	0.51	1.000
**Ch520**	50	5	62.50	3.79	0.56	0.49	0.791	19	4	50.00	3.91	0.63	0.60	0.228	41	7	87.50	4.67	0.51	0.57	**<0.001**
**Ch623**	50	17	51.52	12.04	0.96	0.92	0.025	19	11	33.33	8.73	0.74	0.85	0.516	41	21	63.64	13.07	0.98	0.93	0.569
**Ch10105**	50	4	44.44	2.69	0.18	0.17	1.000	19	4	44.44	3.69	0.47	0.46	0.545	41	5	55.56	3.21	0.27	0.28	0.364
**Ch10857**	50	8	47.06	5.62	0.58	0.70	0.389	19	6	35.29	5.32	0.74	0.65	0.487	41	8	47.06	6.51	0.78	0.75	0.773
**Ch13222**	50	2	33.33	1.99	0.30	0.26	0.046	19	3	50.00	2.36	0.16	0.15	1.000	41	4	66.67	2.45	0.15	0.14	1.000
**Ch18085**	50	10	71.43	7.23	0.72	0.83	0.121	19	9	64.29	7.46	0.89	0.83	0.534	41	7	50.00	6.59	0.78	0.82	0.999
**Ch2931**	50	11	64.71	7.55	0.80	0.83	0.553	19	8	47.06	7.06	0.63	0.82	0.574	41	9	52.94	6.72	0.63	0.81	0.249
**Ch4796**	50	13	72.22	9.41	0.96	0.88	0.999	19	9	50.00	7.77	0.84	0.86	1.000	41	13	72.22	9.69	0.98	0.88	1.000
**Ch10441**	50	8	34.78	5.72	0.62	0.64	0.855	19	8	34.78	6.19	0.84	0.73	0.102	41	7	30.43	5.15	0.46	0.62	**<0.001**
**Ch11230**	50	6	42.86	4.86	0.64	0.70	0.811	19	6	42.86	5.11	0.63	0.71	0.884	41	7	50.00	5.55	0.71	0.73	0.698
**Ch11483**	50	8	53.33	5.34	0.52	0.54	0.772	19	6	40.00	4.42	0.47	0.58	**<0.001**	41	6	40.00	4.06	0.56	0.54	0.543
**Ch17977**	50	8	53.33	5.08	0.58	0.59	0.082	19	4	26.67	3.70	0.58	0.54	0.690	41	6	40.00	4.47	0.56	0.63	0.419
**Ch19846**	50	9	75.00	6.60	0.74	0.81	0.999	19	8	66.67	6.63	0.89	0.80	0.075	41	10	83.33	7.17	0.80	0.81	0.996
**Ch24332**	50	8	80.00	4.91	0.68	0.65	1.000	19	4	40.00	3.52	0.42	0.63	0.011	41	6	60.00	4.31	0.76	0.64	0.666
**Ch25478**	50	8	50.00	4.53	0.66	0.62	0.975	19	6	37.50	4.13	0.47	0.55	1.000	41	7	43.75	4.47	0.68	0.64	0.542

	HB14							AB14							RS96						
**Locus**	***n***	N_A	**%**	A_R	H_O	H_E	***pHWE***	***n***	N_A	**%**	A_R	H_O	H_E	***pHWE***	***n***	N_A	**%**	A_R	H_O	H_E	***pHWE***
**Ch126**	82	5	71.43	2.81	0.57	0.53	0.017	25	3	42.86	2.84	0.60	0.55	1.000	91	3	42.86	2.62	0.55	0.52	0.331
**Ch520**	82	7	87.50	4.23	0.57	0.54	0.391	25	5	62.50	3.76	0.48	0.47	**<0.001**	91	7	87.50	4.04	0.58	0.52	0.069
**Ch623**	82	21	63.64	12.94	0.87	0.93	0.946	25	15	45.45	11.64	0.96	0.91	1.000	91	23	69.70	13.70	0.88	0.93	0.988
**Ch10105**	82	4	44.44	3.18	0.27	0.27	0.159	25	4	44.44	2.33	0.12	0.12	1.000	91	5	55.56	3.27	0.25	0.26	0.600
**Ch10857**	82	8	47.06	5.38	0.57	0.69	0.931	25	8	47.06	6.06	0.76	0.73	0.480	91	11	64.71	6.70	0.71	0.76	0.625
**Ch13222**	82	4	66.67	2.50	0.20	0.20	1.000	25	2	33.33	1.96	0.16	0.21	0.335	91	3	50.00	2.20	0.16	0.18	0.203
**Ch18085**	82	9	64.29	7.30	0.83	0.83	0.996	25	7	50.00	6.24	0.84	0.81	0.997	91	11	78.57	7.69	0.89	0.84	1.000
**Ch2931**	82	12	70.59	6.92	0.73	0.81	0.795	25	9	52.94	7.06	0.80	0.83	**0.008**	91	12	70.59	6.85	0.68	0.79	0.630
**Ch4796**	82	15	83.33	9.70	0.89	0.88	0.166	25	10	55.56	7.67	0.88	0.85	**<0.001**	91	14	77.78	9.73	0.91	0.89	1.000
**Ch10441**	82	10	43.48	5.54	0.48	0.64	0.010	25	7	30.43	5.48	0.68	0.60	0.973	91	11	47.83	6.12	0.67	0.71	0.827
**Ch11230**	82	8	57.14	5.14	0.67	0.72	0.978	25	6	42.86	4.59	0.68	0.70	**0.004**	91	7	50.00	4.79	0.70	0.68	0.831
**Ch11483**	82	10	66.67	4.60	0.54	0.54	0.543	25	6	40.00	4.71	0.68	0.58	0.308	91	9	60.00	4.82	0.55	0.55	0.966
**Ch17977**	82	7	46.67	4.71	0.62	0.61	0.777	25	7	46.67	5.08	0.76	0.63	0.021	91	9	60.00	5.22	0.62	0.62	0.940
**Ch19846**	82	10	83.33	7.23	0.78	0.82	0.477	25	7	58.33	6.13	0.68	0.79	0.088	91	10	83.33	6.88	0.87	0.80	0.997
**Ch24332**	82	8	80.00	4.72	0.60	0.65	0.315	25	5	50.00	3.57	0.64	0.57	0.422	91	10	100.00	4.87	0.64	0.67	0.564
**Ch25478**	82	9	56.25	3.70	0.50	0.58	0.208	25	6	37.50	4.21	0.68	0.62	**0.005**	91	9	56.25	4.37	0.63	0.61	0.970

	RS97						
**Locus**	***n***	**N_A**	**%**	**A_R**	**H_O**	**H_E**	***pHWE***
**Ch126**	84	3	42.86	2.60	0.54	0.53	1.000
**Ch520**	84	6	75.00	3.88	0.42	0.48	0.393
**Ch623**	84	24	72.73	13.54	0.93	0.93	0.786
**Ch10105**	84	4	44.44	3.12	0.26	0.25	0.759
**Ch10857**	84	10	58.82	6.29	0.73	0.76	0.065
**Ch13222**	84	3	50.00	2.27	0.23	0.23	0.311
**Ch18085**	84	10	71.43	7.83	0.86	0.83	1.000
**Ch2931**	84	9	52.94	6.44	0.68	0.79	0.883
**Ch4796**	84	15	83.33	9.44	0.83	0.87	0.398
**Ch10441**	84	10	43.48	6.04	0.60	0.64	0.517
**Ch11230**	84	8	57.14	4.72	0.71	0.68	**0.002**
**Ch11483**	84	8	53.33	4.19	0.55	0.51	0.740
**Ch17977**	84	8	53.33	5.09	0.57	0.62	0.377
**Ch19846**	84	10	83.33	6.88	0.82	0.80	0.870
**Ch24332**	84	7	70.00	4.35	0.68	0.64	1.000
**Ch25478**	84	8	50.00	4.01	0.60	0.60	0.111

Table S2 Primer sequences and amplification conditions used to amplify 16 EST-linked microsatellite loci in *Pleuragramma antarctica*. Locus names, repeat motifs, primer pair sequences, fluorescent dye of forward primer, size range of fragments in base pairs, annealing temperature for single-locus amplification, and the respective multiplex reaction set are shown (adapted from Agostini *et al.*[2]).

Locus	Motif	Primer sequences (5' - 3')	Dye	Size range	T^a (˚C)	Locus set for multiplexing
Ch10105[b]	$(CTG)_{12}$	**F:** TGC CTT GGT TAG GAT TAA ACG T	6-FAM	96 - 131	57	1
		**R:** AGA AGT GCT CCA TCA AGT CCA				
Ch10441[b]	$(CA)_{10}$	**F:** GTC CTT ACC TGG CAG TGC AGA	PET	110 - 162	60	2
		**R:** GCA CAT CAG TCC AAA ATC GGT G				
Ch10857[b]	$(CA)_9$	**F:** GCT TAA ATC ACC ATG TGC CCA	6-FAM	161 - 195	57	1
		**R:** TGG ACA TGA ATG TAC CAA AAC G				
Ch11230[b]	$(GA)_7$	**F:** ATC ACA CAG CTG ACT GGG GCT	NED	90 - 116	60	2
		**R:** TGA GAC TCG TCC GCG AAT GGA				
Ch11483[b]	$(TAA)_7$	**F:** ATC AAA CTG AAC AGC CTG TGT	NED	124 - 168	57	2
		**R:** TGG AGA ACA GGA ATG GAC AGA				
Ch126[c]	$(AAT)_7$	**F:** CGG TTT TTA TGC ATG TTG CCA	NED	264 - 282	56	1
		**R:** ACT GCT CAT TCA CAC TGG TTC				
Ch13222[b]	$(TTG)_6$	**F:** GTC CCA TGG TGA CTG ATA GGT	PET	82 - 98	57	1
		**R:** ACG AGT CAA TGT AAC CGG AAG				
Ch17977[b]	$(GAT)_8$	**F:** TTG ACT GAC AGC TTG GGT GCA	6-FAM	162 - 234	57	2
		**R:** GAA TGA GCC ATG TTG AGC CC				
Ch18085[b]	$(TGA)_6$	**F:** TTT AGG GGT GGC ACA TTG GAC	VIC	98 - 137	57	1
		**R:** ACA AAC CTT CTC CTT GTC TTC T				
Ch19846[b]	$(GT)_{14}$	**F:** CGA CCT TTG AGA AAG GCG GCA	6-FAM	116 - 141	60	2
		**R:** CGA CGT GTA TCA CAA GGG TCA				
Ch24332[b]	$(GCA)_8$	**F:** ATC GGT TGT CGT CCT CCA CAC	6-FAM	67 - 96	60	2
		**R:** GGC TGT TTC TGG ATC AGC GGT				
Ch25478[b]	$(AC)_8$	**F:** ACA GTG TTG TCT TGG AGA GGT	PET	143 - 175	57	2
		**R:** GGA GAG AAG TAC ATG AGG AGG A				
Ch2931[b]	(TCT)8t-$(TTC)_6$	**F:** GGG CTT TCA GGA GCA TCG GGA	VIC	79 - 132	60	2
		**R:** ACT TGA ACC TGA CGT GGC AAC				
Ch4796[b]	$(GAT)_8$	**F:** ACA ATG GTT GGT GAG AGC TCC	VIC	156 - 208	57	2
		**R:** TGA GTT AAG CAG AAC AAG TGC				
Ch520[b]	$(TC)_7$	**F:** GGA ACA ACT TGA GCC AAG ACA	6-FAM	259 - 273	57	1
		**R:** CCA TAA AAG TGC ATC ATC GCT				
Ch623[c]	$(AAC)_7$	**F:** GCT GTT TGA TTC CCT CGT GAG G	PET	174 - 295	62	1
		**R:** AAA AGT GGT CCT CCG CTG CAG T				

[a] Reported annealing temperature refers to single-locus PCR. Loci were amplified together in two multiplex reactions. Multiplex PCR reaction volume was 10 µL, containing 1x QIAGEN Multiplex PCR Master mix (HotStartTaq DNA Polymerase, Multiplex PCR Buffer, dNTP Mix; QIAGEN, Hilden, Germany), 0.2 µM primer mix and 100 ng of genomic DNA. The PCR amplification profile for all loci consisted of: (1) an initial activation step of 15 min at 95 ˚C; (2) 30 cycles of denaturation at 94 ˚C for 30 s, annealing at 57 ˚C for 90 s and extension at 72 ˚C for 60 s; and (3) a final extension of 30 min at 60 ˚C.

[b] Molecular Ecology Resources Primer Development Consortium *et al.*[3]

[c] Molecular Ecology Resources Primer Development Consortium *et al.*[4]

Table S3 Analysis of Molecular Variance (AMOVA) among 19 populations of *Pleuragramma antarctica*, among six groups based on geographic origin, and among individuals within populations. Global results for fixation indices F_{ST}, F_{SC}, and F_{CT} reported in upper table, results by locus reported in lower table.

**Source of variation**	***df***	**Proportion of variation**	***P*-value**	**Fixation Indices**	
Among groups	5	0.16	0.00218 ± 0.00050	F_{ST}	0.00161
Among populations within groups	13	0.00	0.63050 ± 0.00429	F_{SC}	0.00002
Among individuals within populations	2115	99.84	0.00644 ± 0.00087	F_{CT}	0.00159

**Locus**	F_{SC}	***P*-value**	F_{ST}	***P*-value**	F_{SC}	***P*-value**
Ch126	0.00222	0.21436	0.00322	0.11287	0.00100	0.31168
Ch520	0.00272	0.14782	0.00583	0.01525	0.00311	0.16238
Ch623	-0.00118	0.93178	-0.00083	0.93248	0.00035	0.18139
Ch10105	0.00264	0.16455	0.00529	0.02604	0.00266	0.18634
Ch10857	0.00030	0.50772	0.00173	0.24465	0.00143	0.14436
Ch13222	0.00253	0.21772	0.00267	0.19158	0.00014	0.44020
Ch18085	-0.00079	0.67248	-0.00131	0.81139	-0.00051	0.79634
Ch2931	0.00256	0.18356	0.00231	0.16030	-0.00025	0.57743
Ch4796	-0.00143	0.84980	-0.00073	0.78752	0.00070	0.38485
Ch10441	0.00197	0.24109	0.00197	0.19099	0.00000	0.42762
Ch11483	0.00111	0.32614	-0.00120	0.65604	-0.00231	0.99822
Ch17977	-0.00251	0.87376	-0.00239	0.93010	0.00012	0.46446
Ch19846	-0.00047	0.61356	0.01231	0.00000	0.01277	0.00059
Ch24332	-0.00156	0.71347	-0.00190	0.84584	-0.00034	0.64149
Ch25478	-0.00297	0.87149	0.00042	0.50792	0.00339	0.01970

Table S4 Results of power analysis for the Fisher's and Chi-square tests by using POWSIM ver. 4.1[5]. To assess the statistical power of the 15 microsatellites to detect F_{ST} values ≤ 0.01, observed sample sizes and allele frequencies were taken from the 19 sampling locations using 1000 replicates. Following the POWSIM manual, several combinations of N_e (1000 – 10000) and generations (t = 2 – 201) were tested to account for the variability in N_e estimation. Power is given in % for Fisher's and Chi-square tests.

N_e	F_{ST} = 0.01			F_{ST} = 0.005			F_{ST} = 0.0025			F_{ST} = 0.001		
	t	Fisher	Chi-square	t	Fisher	Chi-square	t	Fisher	Chi-square	t	Fisher	Chi-square
**1000**	*20*	100	100	*10*	100	100	*5*	100	100	*2*	96.2	95.3
**5000**	*100*	100	100	*50*	100	100	*25*	100	100	*10*	96.4	94.1
**10000**	*201*	100	100	*100*	100	100	*50*	100	100	*20*	97.4	94.6

Table S5 Comparison of tests of genetic differentiation among *Pleuragramma antarctica* samples from the Antarctic Peninsula included in the analysis of Agostini *et al.*[2]. Pairwise F_{ST} estimates (above the diagonal) and corresponding *P*-values (below the diagonal) are shown. Values in bold were significant after correction for multiple tests as implemented in SGoF+. Comparisons within the same geographic region are delineated with dotted lines. **a** Original pairwise F_{ST} analysis adapted from Table 3 in Agostini *et al.*[2] (threshold for significance with 36 comparisons $P = 0.0180$). **b** Pairwise F_{ST} analysis of samples from Agostini *et al.*[2] using 15 loci after the removal of locus Ch11230 (threshold for significance with 36 comparisons $P = 0.0160$). Values shaded in light gray indicate gains of significance compared to the original analysis[2]. Values shaded in dark gray indicate losses of significance compared to the original analysis[2]. Numbers in parentheses adjacent to group names indicate sample size (*n*). Sampling location acronyms are as in Table 1.

a

**Original Analysis**	**Western Antarctic Peninsula**					**Northern Antarctic Peninsula**			**Larsen Bay**
	**CI10 (60)**	**MB01 (28)**	**MB02 (49)**	**MB10 (60)**	**MB11 (83)**	**JI07 (34)**	**JI10 (148)**	**JI12 (54)**	**LB07 (46)**
**CI10**	-	-0.0011	-0.0008	0.0007	-0.0001	-0.0009	**0.0065**	**0.0102**	0.0061
**MB01**	0.7027	-	-0.0015	-0.0026	-0.0015	0.0017	0.0057	**0.0086**	0.0063
**MB02**	0.6216	0.7207	-	0.0005	0.0009	0.0007	**0.0037**	**0.0093**	0.0035
**MB10**	0.3333	0.8018	0.4054	-	0.0004	-0.0028	0.0017	0.0062	0.0029
**MB11**	0.5496	0.6757	0.2342	0.2793	-	-0.0004	**0.0041**	**0.0060**	**0.0089**
**JI07**	0.7838	0.2973	0.3874	0.8559	0.5315	-	-0.0015	0.0008	0.0001
**JI10**	**< 0.0001**	0.0180	**< 0.0001**	0.0541	**< 0.0001**	0.8108	-	0.0020	0.0020
**JI12**	**< 0.0001**	**0.0090**	**< 0.0001**	0.0270	**< 0.0001**	0.3874	0.0991	-	**0.0057**
**LB07**	0.0180	0.0991	0.0811	0.0451	**< 0.0001**	0.4955	0.0991	**0.0090**	-

b

**15 loci w/o Ch11230**	**Western Antarctic Peninsula**					**Northern Antarctic Peninsula**			**Larsen Bay**
	**CI10 (60)**	**MB01 (28)**	**MB02 (49)**	**MB10 (60)**	**MB11 (83)**	**JI07 (34)**	**JI10 (148)**	**JI12 (54)**	**LB07 (46)**
**CI10**	-	0.0021	0.0003	0.0003	-0.0010	-0.0006	**0.0068**	**0.0111**	**0.0057**
**MB01**	0.2793	-	0.0019	0.0002	0.0004	-0.0001	0.0041	0.0059	0.0042
**MB02**	0.4454	0.2315	-	0.0007	0.0002	0.0012	**0.0035**	**0.0084**	0.0041
**MB10**	0.4566	0.4512	0.3341	-	0.0009	-0.0020	0.0027	**0.0085**	0.0025
**MB11**	0.7577	0.3860	0.3902	0.2479	-	0.0002	**0.0051**	**0.0082**	**0.0083**
**JI07**	0.6186	0.4475	0.2680	0.8035	0.3916	-	-0.0010	0.0019	0.0008
**JI10**	**< 0.0001**	0.0556	**0.0160**	0.0234	**< 0.0001**	0.7230	-	0.0025	0.0019
**JI12**	**< 0.0001**	0.0444	**0.0006**	**0.0003**	**0.0001**	0.2279	0.0514	-	**0.0060**
**LB07**	**0.0160**	0.1147	0.0441	0.1259	**0.0002**	0.3588	0.1176	**0.0120**	-

Table S6 Standard length (SL) analysis. **a** SL-derived groupings independent of sampling location. **b – g** Pairwise F_{ST} estimates (above the diagonal) and corresponding *P*-values (below the diagonal) are shown. Values in bold were significant after correction for multiple tests as implemented in SGoF+[1]. Group numbers correspond to respective groupings in **a**. Group SL range (cm) in parentheses to the left of each matrix. Sample size (*n*) in parentheses above each matrix.

a

SL Grouping 1

**Groups**	**cm**	***n***
1	3 - 4	3
2	4 - 5	46
3	5 - 6	14
4	6 - 7	16
5	7 - 8	5
6	8 - 9	14
7	9 - 10	56
8	10 - 11	83
9	11 - 12	81
10	12 - 13	58
11	13 - 14	30
12	14 - 15	45
13	15 - 16	78
14	16 - 17	72
15	17 - 18	32
16	18 - 19	23
17	19 - 20	7
18	20 - 21	4
19	21 - 22	4

SL Grouping 2

**Groups**	**cm**	***n***
1	3 - 5	49
2	5 - 7	30
3	7 - 9	19
4	9 - 11	139
5	11 - 13	139
6	13 - 15	75
7	15 - 17	150
8	17 - 19	55
9	19 - 21	11
10	21 - 23	4

SL Grouping 3

**Groups**	**cm**	***n***
1	3 - 6	63
2	6 - 9	35
3	9 -12	220
4	12 - 15	133
5	15 - 18	182
6	18 - 21	34
7	21 - 24	4

SL Grouping 4

**Groups**	**cm**	***n***
1	3 - 7	79
2	7 - 11	158
3	11 - 15	214
4	15 - 19	205
5	19 - 23	15

SL Grouping 5

**Groups**	**cm**	***n***
1	3 - 8	84
2	8 - 13	292
3	13 - 18	257
4	18 - 23	38

SL Grouping 6

**Groups**	**cm**	***n***
1	3 - 9	98
2	9 - 15	353
3	15 - 22	220

b SL Grouping 1

(cm) \ (*n*)	1 (3)	2 (46)	3 (14)	4 (16)	5 (5)	6 (14)	7 (56)	8 (83)	9 (81)	10 (58)	11 (30)	12 (45)	13 (78)	14 (72)	15 (32)	16 (23)	17 (7)	18 (4)	19 (4)
**1 (3 - 4)**	-	0.0486	0.0163	0.0515	0.0097	0.0454	0.0509	0.0575	**0.0457**	0.0571	0.0407	0.0563	0.0395	0.0472	0.0485	0.0541	0.0559	-0.0081	0.0330
**2 (4 - 5)**	0.0180	-	0.0039	0.0047	-0.0016	-0.0016	0.0031	0.0018	0.0012	0.0042	0.0003	0.0004	0.0020	0.0012	0.0019	0.0025	0.0016	0.0083	-0.0059
**3 (5 - 6)**	0.3153	0.2613	-	0.0070	-0.0037	0.0081	0.0120	0.0047	0.0023	0.0070	-0.0028	0.0092	0.0042	0.0035	-0.0007	-0.0015	0.0196	-0.0057	-0.0101
**4 (6 - 7)**	0.0631	0.1351	0.1892	-	-0.0240	0.0012	0.0033	-0.0012	-0.0031	-0.0013	-0.0020	0.0025	-0.0008	0.0031	-0.0031	**0.0053**	0.0270	0.0107	-0.0103
**5 (7 - 8)**	0.6306	0.5405	0.5405	0.9550	-	-0.0185	-0.0173	-0.0115	-0.0134	-0.0200	-0.0094	-0.0095	-0.0104	-0.0129	-0.0113	-0.0040	0.0068	-0.0357	-0.0241
**6 (8 - 9)**	0.1261	0.7387	0.1351	0.3694	0.9730	-	0.0024	-0.0055	-0.0029	-0.0003	-0.0011	-0.0034	-0.0046	-0.0006	-0.0036	-0.0007	-0.0113	-0.0153	-0.0196
**7 (9 - 10)**	0.0541	0.1081	0.0180	0.2793	0.9640	0.3514	-	0.0030	0.0016	-0.0001	0.0053	0.0024	0.0024	0.0020	-0.0002	0.0064	0.0048	0.0028	-0.0008
**8 (10 - 11)**	0.0180	0.1532	0.1351	0.6667	0.8919	0.9369	0.0541	-	-0.0019	-0.0003	-0.0008	-0.0013	-0.0013	0.0005	-0.0032	-0.0012	0.0058	0.0042	-0.0093
**9 (11 - 12)**	0.0180	0.2072	0.2613	0.8378	0.9279	0.7568	0.1261	0.9369	-	-0.0002	-0.0010	-0.0014	-0.0021	-0.0004	-0.0013	-0.0009	0.0033	0.0020	-0.0120
**10 (12 - 13)**	**< 0.0001**	0.0090	0.0901	0.6667	0.9820	0.5045	0.5676	0.5766	0.6126	-	0.0022	0.0021	0.0023	0.0004	0.0003	0.0028	0.0050	0.0061	-0.0112
**11 (13 - 14)**	0.0631	0.5045	0.7658	0.6487	0.8649	0.6487	0.0541	0.5946	0.6306	0.2072	-	0.0015	0.0001	0.0037	0.0004	-0.0048	0.0119	0.0063	-0.0133
**12 (14 - 15)**	0.0360	0.4955	0.0451	0.3243	0.8018	0.8108	0.2072	0.7568	0.7928	0.1622	0.3153	-	-0.0013	0.0026	-0.0012	0.0004	0.0027	0.0104	-0.0132
**13 (15 - 16)**	0.0541	0.1261	0.1351	0.5676	0.8559	0.8829	0.0541	0.8288	0.9550	0.1081	0.4414	0.7748	-	0.0004	-0.0010	0.0003	0.0023	0.0019	-0.0068
**14 (16 - 17)**	0.0180	0.3063	0.2793	0.2162	0.9460	0.5946	0.1441	0.2342	0.6126	0.3964	0.0901	0.1081	0.3964	-	0.0018	0.0016	0.0047	0.0077	0.0012
**15 (17 - 18)**	0.0270	0.3243	0.5946	0.7478	0.8919	0.8378	0.5496	0.9279	0.7658	0.4505	0.4505	0.6577	0.7748	0.3514	-	0.0027	0.0097	0.0050	-0.0152
**16 (18 - 19)**	0.0270	0.2252	0.6036	0.1441	0.7297	0.5676	0.0631	0.5766	0.5676	0.1982	0.9369	0.3874	0.4505	0.2613	0.2523	-	0.0106	0.0174	-0.0139
**17 (19 - 20)**	0.1441	0.5315	0.0901	**< 0.0001**	0.4324	0.9460	0.3063	0.2703	0.3694	0.3153	0.1712	0.4505	0.3964	0.3423	0.1802	0.1351	-	0.0040	-0.0040
**18 (20 - 21)**	0.7568	0.3333	0.6577	0.2162	0.9550	0.8739	0.4865	0.4505	0.6216	0.3604	0.3243	0.2252	0.5586	0.3964	0.3694	0.1261	0.5586	-	-0.0083
**19 (21 - 22)**	0.4324	0.7478	0.7838	0.7928	0.9279	0.9640	0.6396	0.7387	0.7658	0.7838	0.8829	0.9099	0.6847	0.4865	0.8829	0.8108	0.7478	0.6126	-

c SL Grouping 2

(cm) \ (*n*)	1 (49)	2 (30)	3 (19)	4 (139)	5 (139)	6 (75)	7 (150)	8 (55)	9 (11)	10 (4)
**1 (3 - 5)**	-	0.0016	0.0016	0.0019	0.0025	0.0002	0.0012	0.0016	0.0024	-0.0061
**2 (5 - 7)**	0.2523	-	0.0014	0.0013	-0.0011	0.0003	0.0005	-0.0028	0.0134	-0.0118
**3 (7 - 9)**	0.3874	0.3694	-	-0.0025	-0.0020	-0.0010	-0.0014	-0.0011	-0.0089	-0.0160
**4 (9 - 11)**	0.1982	0.2793	0.8649	-	-0.0011	-0.0003	-0.0002	-0.0018	0.0029	-0.0065
**5 (11 - 13)**	0.0541	0.7117	0.7297	0.9460	-	-0.0002	-0.0002	-0.0007	0.0029	-0.0115
**6 (13 - 15)**	0.4505	0.4505	0.6487	0.6306	0.5856	-	0.0006	-0.0020	0.0062	-0.0135
**7 (15 - 17)**	0.2252	0.3964	0.6667	0.6216	0.6306	0.3243	-	-0.0001	0.0031	-0.0030
**8 (17 - 19)**	0.2252	0.8919	0.5676	0.9369	0.6396	0.9189	0.6487	-	0.0083	-0.0152
**9 (19 - 21)**	0.4595	0.0721	0.9189	0.2973	0.3243	0.1171	0.3063	0.0991	-	-0.0051
**10 (21 - 23)**	0.7838	0.8198	0.9820	0.6757	0.8018	0.9009	0.5496	0.9099	0.6036	-

d SL Grouping 3

(cm) \ (*n*)	1 (63)	2 (35)	3 (220)	4 (133)	5 (182)	6 (34)	7 (4)
**1 (3 - 6)**	-	0.0044	0.0022	0.0020	0.0012	-0.0002	-0.0072
**2 (6 - 9)**	0.0451	-	-0.0010	-0.0008	-0.0001	0.0015	-0.0124
**3 (9 - 12)**	0.0270	0.8108	-	-0.0009	-0.0007	-0.0012	-0.0082
**4 (12 - 15)**	0.0901	0.6847	0.9369	-	0.0002	-0.0013	-0.0129
**5 (15 - 18)**	0.1892	0.6036	0.9460	0.2973	-	-0.0005	-0.0051
**6 (18 - 21)**	0.5856	0.3243	0.7838	0.6757	0.5946	-	-0.0135
**7 (21 - 24)**	0.6757	0.8739	0.7207	0.9279	0.6216	0.8649	-

e SL Grouping 4

(cm) \ (*n*)	1 (79)	2 (158)	3 (214)	4 (205)	5 (15)
**1 (3 - 7)**	-	0.0015	0.0004	0.0003	0.0033
**2 (7 - 11)**	0.1171	-	-0.0006	-0.0004	-0.0001
**3 (11 - 15)**	0.3423	0.8469	-	-0.0002	0.0005
**4 (15 - 19)**	0.4595	0.7478	0.7748	-	0.0025
**5 (19 - 23)**	0.2613	0.5315	0.3784	0.2883	-

f SL Grouping 5

(cm) \ (*n*)	1 (84)	2 (292)	3 (257)	4 (38)
**1 (3 - 8)**	-	0.0007	-0.0002	0.0008
**2 (8 - 13)**	0.1802	-	-0.0002	-0.0007
**3 (13 - 18)**	0.5225	0.7658	-	-0.0004
**4 (18 - 23)**	0.4144	0.6487	0.5496	-

g SL Grouping 6

(cm) \ (*n*)	1 (98)	2 (353)	3 (220)
**1 (3 - 9)**	-	0.0002	-0.0003
**2 (9 - 15)**	0.3964	-	-0.0003
**3 (15 - 22)**	0.7838	0.7748	-

Table S7 Genetic differentiation among *Pleuragramma antarctica* samples including small and large size clusters from Joinville Island 2010. Pairwise F_{ST} estimates (above the diagonal) and corresponding *P*-values (below the diagonal) are shown. Values in bold were significant after correction for multiple tests as implemented in SGoF+[1] (threshold for significance with 190 comparisons $P = 0.0453$). Comparisons within the same geographic region are delineated with dotted lines. Values shaded in light gray indicate gains of significance compared to the original analysis[2]. Values shaded in dark gray indicate losses of significance compared to the original analysis[2]. Numbers in parentheses adjacent to group names indicate sample size (*n*). Sampling location acronyms are as in Table 1. JI10S, Joinville Island 2010 small cluster; JI10L, Joinville Island 2010 large cluster; SOI, South Orkney Islands.

	Western Antarctic Peninsula (WAP)					Northern Antarctic Peninsula (NAP)					SOI	Larsen Bay (LB)		Weddell Sea (WS)					Ross Sea (RS)	
	CI10 (60)	MB01 (28)	MB02 (49)	MB10 (60)	MB11 (83)	JI07 (34)	JI10S (68)	JI10L (72)	JI12 (54)	SSI96 (14)	SOI11 (47)	LB07 (46)	LB11 (52)	FT14 (50)	HB89 (19)	HB91 (41)	HB14 (82)	AB14 (25)	RS96 (91)	RS97 (84)
**CI10**	-	-0.0008	-0.0002	0.0007	-0.0008	-0.0033	**0.0061**	**0.0048**	**0.0043**	-0.0044	**0.0100**	**0.0054**	0.0021	0.0002	0.0067	0.0016	0.0001	-0.0012	**0.0049**	0.0024
**MB01**	0.6810	-	-0.0004	-0.0022	-0.0016	0.0006	**0.0070**	0.0043	0.0047	0.0016	**0.0125**	0.0057	0.0057	0.0007	0.0051	0.0037	0.0015	0.0008	0.0043	0.0042
**MB02**	0.5787	0.5126	-	0.0015	0.0010	-0.0013	**0.0041**	0.0016	0.0039	-0.0041	**0.0103**	0.0021	0.0006	0.0010	0.0062	0.0004	-0.0003	-0.0036	0.0023	**0.0043**
**MB10**	0.3758	0.7993	0.2069	-	0.0009	-0.0037	**0.0032**	0.0006	**0.0037**	-0.0012	**0.0091**	0.0027	-0.0005	-0.0000	0.0047	0.0008	-0.0011	-0.0021	0.0002	0.0010
**MB11**	0.7213	0.7356	0.2443	0.2427	-	-0.0008	**0.0061**	**0.0047**	**0.0043**	0.0012	**0.0096**	**0.0094**	**0.0046**	0.0008	0.0060	0.0030	0.0018	-0.0001	**0.0041**	**0.0025**
**JI07**	0.9567	0.4147	0.6655	0.9617	0.6194	-	0.0031	-0.0032	0.0010	-0.0084	**0.0059**	0.0009	-0.0034	-0.0034	0.0054	0.0002	-0.0027	-0.0053	-0.0018	-0.0026
**JI10S**	**0.0041**	**0.0156**	**0.0284**	**0.0399**	**0.0005**	0.1056	-	**0.0040**	0.0034	0.0013	**0.0045**	0.0055	**0.0037**	0.0019	-0.0004	0.0005	0.0021	0.0038	0.0001	**0.0044**
**JI10L**	**0.0096**	0.0691	0.1819	0.3252	**0.0020**	0.9572	**0.0098**	-	0.0024	-0.0042	**0.0042**	0.0003	-0.0018	0.0002	0.0049	0.0032	-0.0008	-0.0018	-0.0000	0.0019
**JI12**	**0.0358**	0.0902	0.0527	**0.0388**	**0.0115**	0.3543	0.0514	0.1143	-	0.0020	0.0013	0.0039	0.0027	-0.0006	0.0053	-0.0015	0.0015	0.0014	0.0020	0.0020
**SSI96**	0.8949	0.3972	0.8144	0.5788	0.3643	0.9734	0.4086	0.8656	0.3729	-	0.0010	0.0012	-0.0057	-0.0062	0.0034	-0.0018	-0.0050	-0.0072	-0.0016	-0.0016
**SOI11**	**0.0006**	**0.0016**	**0.0009**	**0.0006**	**0.0001**	**0.0383**	**0.0265**	**0.0312**	0.3132	0.4789	-	**0.0058**	0.0039	0.0003	0.0066	-0.0001	0.0037	0.0049	**0.0066**	0.0031
**LB07**	**0.0188**	0.0518	0.1708	0.0958	**0.0001**	0.3593	0.0095	0.4401	0.0598	0.4017	**0.0212**	-	0.0020	0.0020	0.0047	**0.0056**	0.0004	0.0004	**0.0033**	0.0033
**LB11**	0.1806	0.0453	0.3619	0.5866	**0.0088**	0.9430	**0.0361**	0.8908	0.1145	0.9241	0.0650	0.1872	-	-0.0012	**0.0098**	-0.0012	-0.0008	-0.0035	0.0016	0.0013
**FT14**	0.5172	0.4127	0.3118	0.4773	0.2877	0.9348	0.1716	0.4705	0.6511	0.9403	0.4792	0.1909	0.7452	-	0.0044	-0.0012	-0.0015	-0.0037	-0.0009	-0.0013
**HB89**	0.0826	0.1752	0.0647	0.1036	0.0501	0.1201	0.5773	0.1046	0.1347	0.3473	0.0970	0.1393	**0.0140**	0.1607	-	0.0047	0.0024	0.0066	0.0017	0.0057
**HB91**	0.2849	0.1499	0.4115	0.3282	0.0648	0.4695	0.4343	0.0736	0.7977	0.6767	0.5613	**0.0245**	0.7272	0.7308	0.1513	-	-0.0006	-0.0031	0.0015	0.0007
**HB14**	0.5501	0.3427	0.6011	0.8075	0.1110	0.9388	0.1104	0.7651	0.2477	0.9273	0.0570	0.4541	0.7321	0.8674	0.3111	0.6730	-	-0.0051	0.0011	-0.0009
**AB14**	0.6987	0.3851	0.8802	0.7514	0.4561	0.9474	0.1031	0.7432	0.3292	0.9054	0.0926	0.4136	0.9021	0.9088	0.0950	0.8464	0.9948	-	0.0011	-0.0004
**RS96**	**0.0038**	0.0557	0.0871	0.4265	**0.0021**	0.8266	0.4733	0.5089	0.1134	0.6513	**0.0019**	**0.0387**	0.1593	0.7272	0.3082	0.2056	0.2117	0.3128	-	**0.0024**
**RS97**	0.0946	0.0675	**0.0183**	0.2533	**0.0392**	0.9226	**0.0061**	0.1035	0.1422	0.6717	0.0702	0.0508	0.2248	0.8198	0.0698	0.3866	0.8077	0.5514	**0.0391**	-

Table S8 Genetic differentiation among *Pleuragramma antarctica* sampling locations from the eastern Weddell Sea. Pairwise F_{ST} estimates (above the diagonal) and corresponding *P*-values (below the diagonal) are shown. Numbers in parentheses adjacent to group names indicate sample size (*n*). FT, Filchner Trough; CL, Coats Land; HB, Halley Bay; AB, Atka Bay.

	**west FT (23)**	**east FT (27)**	**CL (29)**	**HB (52)**	**AB (25)**
**west FT**	-	0.0027	-0.0053	-0.0019	-0.0019
**east FT**	0.2798	-	-0.0015	0.0007	-0.0039
**CL**	0.9602	0.7372	-	-0.0028	-0.0060
**HB**	0.7556	0.4143	0.9051	-	-0.0062
**AB**	0.6489	0.8309	0.9830	0.9954	-

a

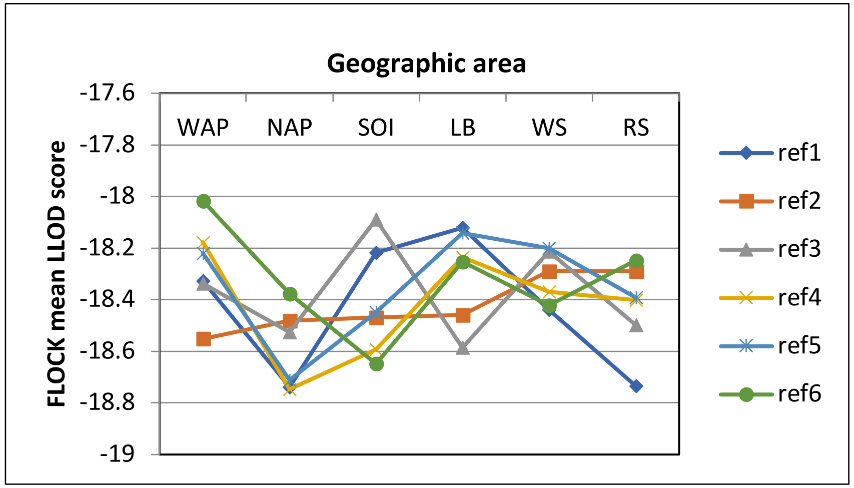

b

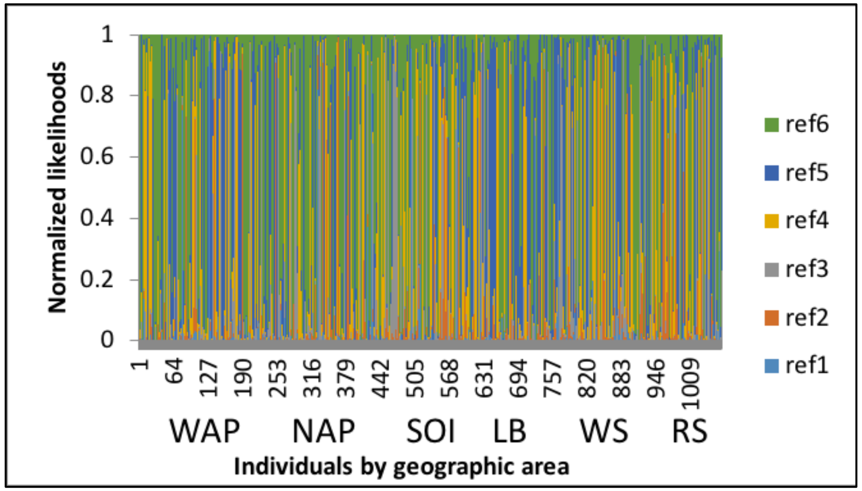

Figure S1 FLOCK clustering results for *Pleuragramma antarctica* populations. **a** Mean LLOD scores are ordered by geographic area. **b** Normalized likelihoods, individuals are ordered by geographic area. ref, reference *k* group cluster (user-define *k* = 6). Initial partition mode by random choice of samples. Number of iterations (re-allocations) per run = 20. Number of runs per parameter *k* = 50. Log likelihood difference (LLOD) threshold = 0 (allocation occurs as soon as there is a highest log-likelihood difference between populations)[6]. Group acronyms are as in Table 1.

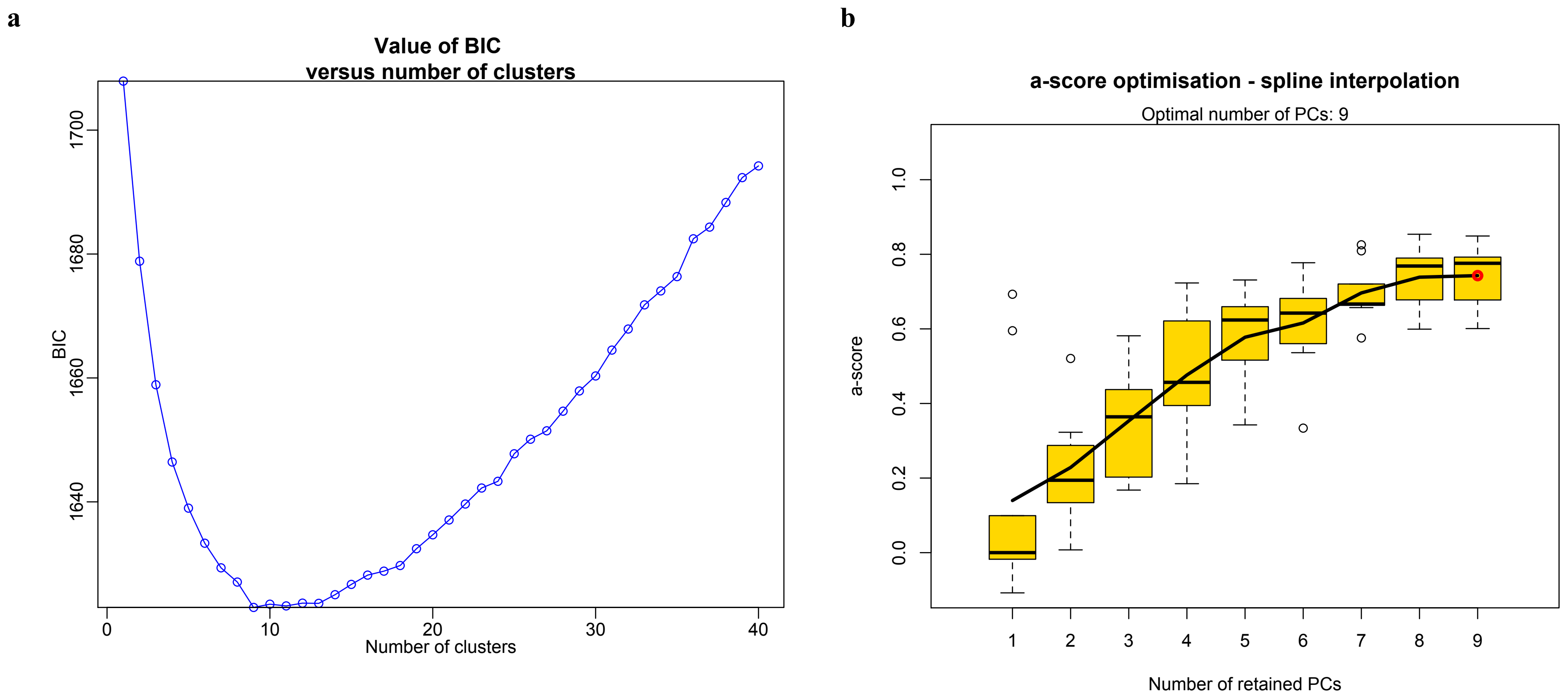

Figure S2 Discriminant analysis of principal components (DAPC) analysis criteria. **a** Bayesian Information Criterion (BIC) plotted for increasing numbers of clusters, indicating the optimal number of clusters at $k = 9$. **b** Plot of a-score optimization based on the *adegenet* R package function *optim.a.*score, used to quantify the trade-off between discrimination power and over-fitting by calculating the difference between the proportion of observed and random discrimination, indicating the optimal number of principal components (PCs) to retain is nine[7].

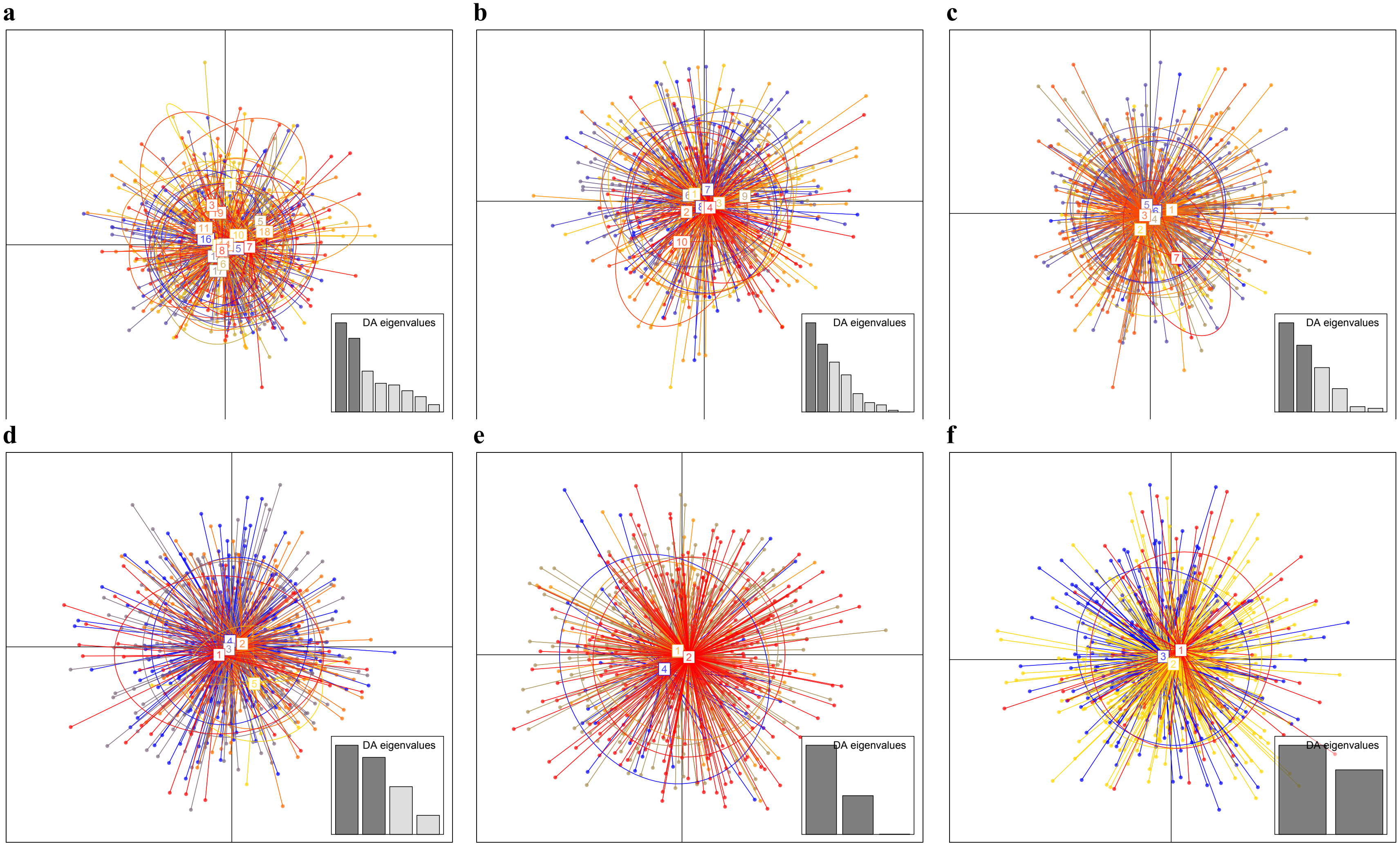

Figure S3 Discriminant analysis of principal components (DAPC) clustering results for *Pleuragramma antarctica* based on location-independent SL groupings (as in Table S6a).

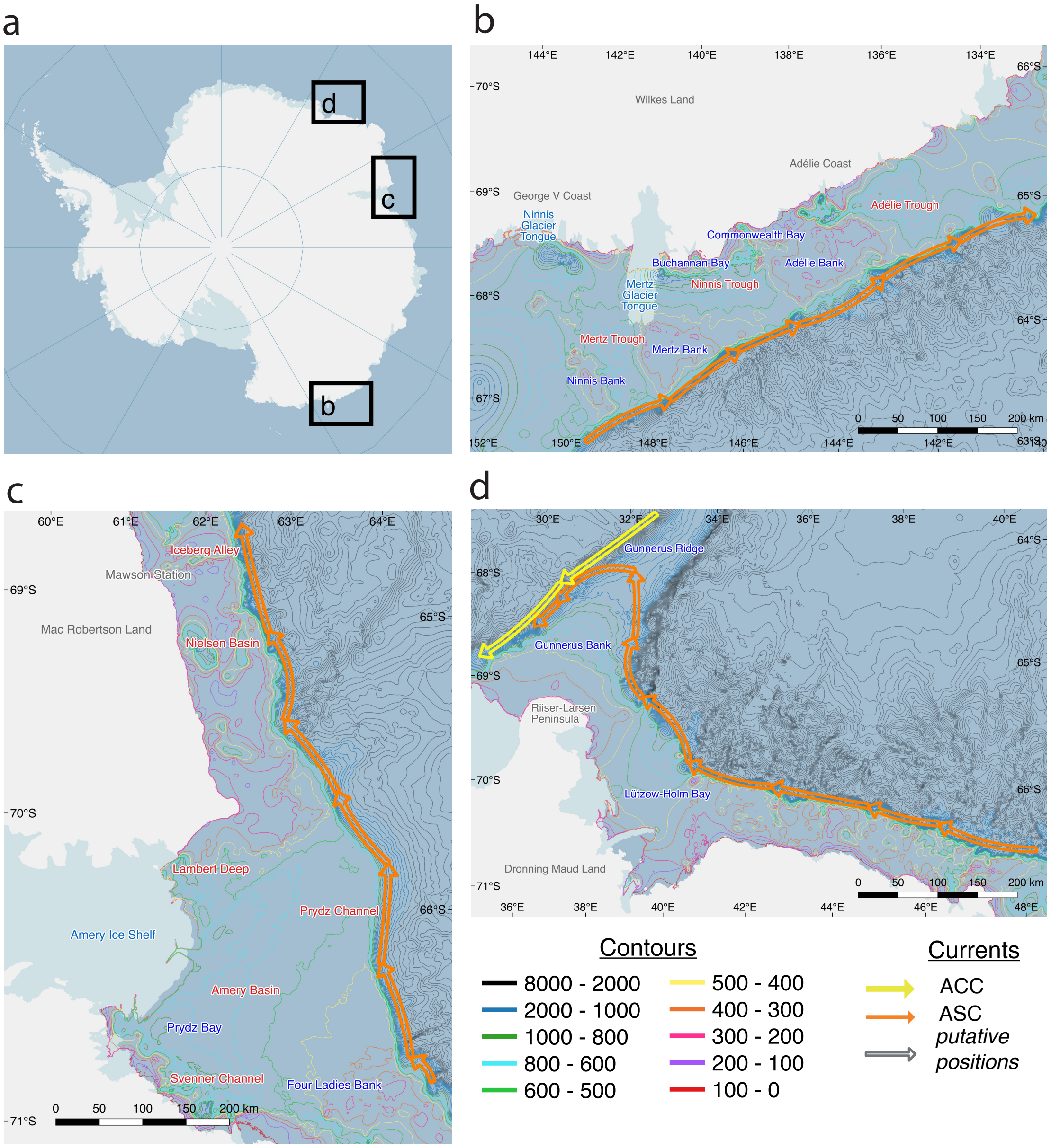

Figure S4 East Antarctica. Arrows indicate suggested positions according to Fig.1 in Ryan *et al.*[8] and Whitworth *et al.*[9] for flow from the ACC and ASC, respectively. **a** Map of the Antarctic. Areas of interest in subsequent maps are indicated in squares and ordered by movement along the ASC. **b** George V and Adélie Coasts. **c** Prydz Bay north to Iceberg Alley. **d** Lützow Bay. Maps created using the Norwegian Polar Institute's Quantarctica 2.0 package[10] in the software QGIS version 2.18.9 http://qgis.osgeo.org[11].

References

1. Carvajal-Rodriguez, A. & de Uña-Alvarez, J. Assessing Significance in High-Throughput Experiments by Sequential Goodness of Fit and q-Value Estimation. *PloS one* **6**, e24700, https://doi.org/10.1371/journal.pone.0024700 (2011).
2. Agostini, C. *et al.* Genetic differentiation in the ice-dependent fish *Pleuragramma antarctica* along the Antarctic Peninsula. *Journal of Biogeography* **42**, 1103-1113, https://doi.org/10.1111/jbi.12497 (2015).
3. Molecular Ecology Resources Primer Development Consortium *et al.* Permanent genetic resources added to Molecular Ecology Resources Database 1 October 2010-30 November 2010. *Molecular ecology resources* **11**, 418-421, https://doi.org/10.1111/j.1755-0998.2010.02970.x (2011).
4. Molecular Ecology Resources Primer Development Consortium *et al.* Permanent Genetic Resources Added to Molecular Ecology Resources Database 1 April 2013-31 May 2013. *Molecular ecology resources* **13**, 966-968, https://doi.org/10.1111/1755-0998.12140 (2013).
5. Ryman, N. & Palm, S. POWSIM: a computer program for assessing statistical power when testing for genetic differentiation. *Molecular Ecology Notes* **6**, 600-602, https://doi.org/10.1111/j.1471-8286.2006.01378.x (2006).
6. Duchesne, P. & Turgeon, J. FLOCK: a method for quick mapping of admixture without source samples. *Molecular ecology resources* **9**, 1333-1344, http://dx.doi.org/10.1111/j.1755-0998.2009.02571.x (2009).
7. Jombart, T. Adegenet: A R package for the multivariate analysis of genetic markers. *Bioinformatics* **24**, 1403-1405, https://doi.org/10.1093/bioinformatics/btn129 (2008).
8. Ryan, S., Schröder, M., Huhn, O. & Timmermann, R. On the warm inflow at the eastern boundary of the Weddell Gyre. *Deep-Sea Research Part I: Oceanographic Research Papers* **107**, 70-81, https://doi.org/10.1016/j.dsr.2015.11.002 (2016).
9. Whitworth, T., Orsi, A. H., Kim, S. J., Nowlin, W. D. & Locarnini, R. A. in *Ocean, Ice, and Atmosphere: Interactions at the Antarctic Continental Margin* 1-27 (American Geophysical Union, 1998).
10. Matsuoka, K., Skoglund, A. & Roth, G. Quantarctica [Data set]. https://doi.org/10.21334/npolar.2018.8516e961 (2018).
11. QGIS Development Team. QGIS Geographic Information System. Open Source Geospatial Foundation Project., https://qgis.org/en/site/index.html (2018).
